# Innovative bioinks for 3D bioprinting: Exploring technological potential and regulatory challenges

**DOI:** 10.1177/20417314241308022

**Published:** 2025-01-20

**Authors:** Vidhi Mathur, Prachi Agarwal, Meghana Kasturi, Varadharajan Srinivasan, Raviraja N Seetharam, Kirthanashri S Vasanthan

**Affiliations:** 1 Manipal Centre for Biotherapeutics Research, Manipal Academy of Higher Education, Manipal, Karnataka, India; 2 Department of Mechanical Engineering, University of Michigan, Dearborn, MI, USA; 3 Department of Civil Engineering, Manipal Institute of Technology, Manipal Academy of Higher Education, Manipal, Karnataka, India

**Keywords:** 3D bioprinting, bioinks, crosslinking, biomedical application

## Abstract

The field of three dimensional (3D) bioprinting has witnessed significant advancements, with bioinks playing a crucial role in enabling the fabrication of complex tissue constructs. This review explores the innovative bioinks that are currently shaping the future of 3D bioprinting, focusing on their composition, functionality, and potential for tissue engineering, drug delivery, and regenerative medicine. The development of bioinks, incorporating natural and synthetic materials, offers unprecedented opportunities for personalized medicine. However, the rapid technological progress raises regulatory challenges regarding safety, standardization, and long-term biocompatibility. This paper addresses these challenges, examining the current regulatory frameworks and the need for updated guidelines to ensure patient safety and product efficacy. By highlighting both the technological potential and regulatory hurdles, this review offers a comprehensive overview of the future landscape of bioinks in bioprinting, emphasizing the necessity for cross-disciplinary collaboration between scientists, clinicians, and regulatory bodies to achieve successful clinical applications.

## Introduction

The techniques that have been widely used to fabricate scaffolds, with the aim of developing 3D structures for tissue engineering, include freeze-drying, leaching, and electrospinning. However, a number of major challenges existed in these methods, which had to do with reproducibility, structural versatility, and the capability to incorporate biological components.

Scaffold fabrication is an important step in tissue engineering as it offers an essential 3D structure that promotes cell proliferation and facilitates tissue regeneration. Although conventional methods like gas foaming and solvent casting are relatively simple, they often need hazardous solvents and fail to provide precise control over pore topologies. On the other hand, methodologies such as rapid prototyping, freeze-drying, electrospinning, and thermally induced phase separation offer enhanced precision with interconnected porosity and controlled pore dimensions. These modifications are essential for optimizing tissue growth and enhancing cell infiltration.^1^

A study by Taherkhani et al. demonstrates that techniques such as solvent casting and particle extraction are useful in producing porous scaffolds for bone tissue engineering.

Advancement in this field in terms of research and developing new products illustrates the significant potential in the healthcare sector as this technique enables the modifications required as per the conditions of the patient and the replication of authentic bone architecture.^2^ Vigani et al. investigated the freeze-drying process, which creates sponge-like scaffolds by eliminating water from polymer solutions. The amount of CaCl_2_ used to make these scaffolds can change the size of their pores.^3^ As a result, structures that are similar to skin gradients are created, which improves the mechanical strength and bioactivity of wound care applications. Another technique that may be used to make nanofiber mats that are comparable to the extracellular matrix is called electrospinning. In their study, Azarsa et al. emphasized the utilization of electrospinning with a mixture of polyvinyl alcohol, polyvinyl chloride, and gelatin. This method enhances the biocompatibility and degradation of the scaffold by adding alginate and decellularized extracellular matrix. Analysis using scanning electron microscopy and histology demonstrated that these scaffolds are capable of facilitating cell penetration and proliferation, making them ideal for use in tissue engineering.^4^

Conventional approaches are capable of producing functioning scaffolds however, they frequently struggle to achieve complicated structures that may be customized with a considerable degree of accuracy. For example, electrospinning has the capability of producing nanofibers; yet, it is still difficult to regulate the alignment of the fibers, the size of the pores, and the dispersion of the material over larger constructions. Although salt leaching can produce porous structures, it does not provide exact control over the design and regularity of the pores.

In the fabrication of intricate scaffolds customized to the specific requirements of each patient, 3D printing is significantly more efficient. It produces structures that resemble the native properties of the tissue by providing meticulous control over structural complexities, porosity, and material layers. These processes permit the incorporation of a wide range of materials into the structure. 3D printing is the preferred technology for regenerative medicine and TE due to its advantages, which include scalability, personalization, and reduced material waste.^5^

Even though it must overcome challenges such as the construction of sophisticated tissue architectures, the realization of vascularization, and the augmentation of printing speed, 3D printing provides significant benefits over conventional methods. These advantages are provided even though it must overcome these challenges. Innovative solutions, such as the utilization of temporary materials for vascularization and sophisticated extrusion techniques, can increase both the structural stability and the survivability of the cells. In addition, the combination of flexible hydrogels with stiff scaffolds increases the mechanical robustness of the material.

These advancements in 3D bioprinting tend to increase the survival of cells as well as the functional mimicry of cells, which ultimately leads to the industry moving forward in the direction of individualized tissue engineering solutions.^6^

The rapid development of additive manufacturing technologies has significantly affected this field, especially in the development of 3D bioprinting capable of handling simultaneously and precisely with living cells, biomaterials, and bioactive molecules to create biologically functional and active constructs. 3D bioprinting solves the disadvantages of conventional processes; for instance, enabling complex scaffold architectures to be precisely designed and reproducibly fabricated.^7^

**Figure 1.**
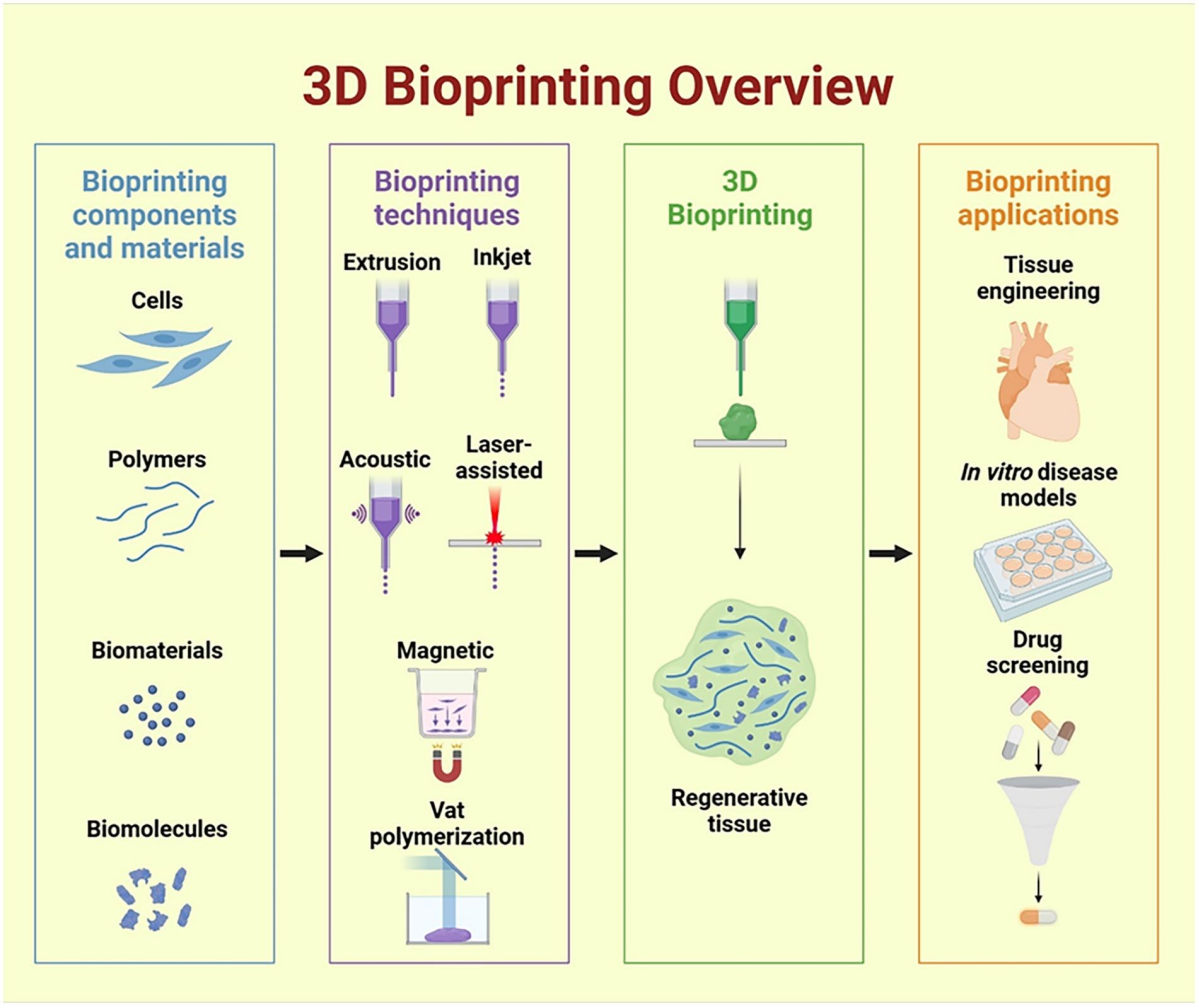
Overview of 3D bioprinting process.

**Figure 2.**
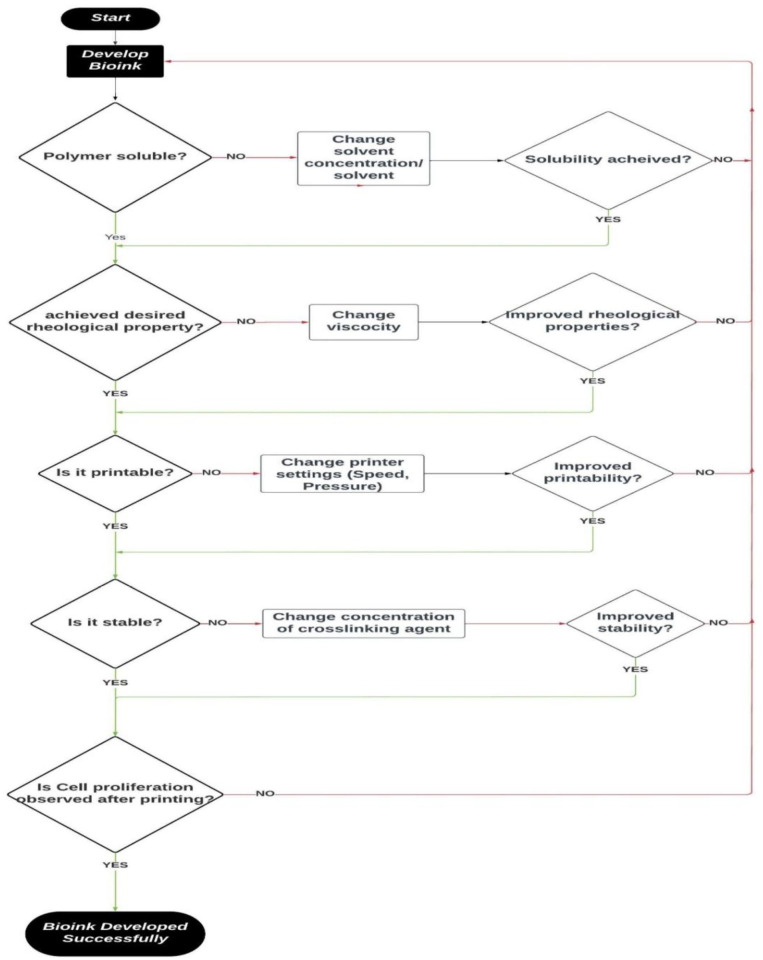
Selection criteria of Bioink for 3D printing. (This work is licensed under a creative comments attribution 3.0 License, Ref.^27^).

3D bioprinting involves different techniques based on ASTM classification which provides a framework to organize 3D bioprinting techniques based on their bioink requirements, principles, construct quality and effect on cell viability and overview of 3D bioprinting technique is shown in Figure 1. ASTM classifies the techniques as extrusion- based, jetting- based and vat polymerization-based technique. Extrusion based bioprinting extrudes bioinks through nozzle. It is the most commonly and widely technique due to its versatility and ability to handle various bioinks having viscosities from 100 to 30,000 mPa.s.^8^ Printability of bioinks using extrusion-based technique depends on the pressure and viscosity. The effectiveness and the quality of the fabricated construct is dependent on two factors: shear stress and crosslinking. Nozzle diameter, extrusion pressure, speed, and bioink’s viscosity decides the magnitude of the shear stress and excessive shear stress can lead to cell damage which in turn will compromise the cell viability. Membrane rupture and apoptosis are the common cause of mechanical damage that excess shear stress causes. Crosslinking is essential to maintain stability and mechanical strength of the construct once printed and it is important to balance the shear stress and crosslinking to ensure maximum cell viability. By selecting the correct parameters, bioink formulation, cell survival and functionality in the constructs can increase and enhance.^9^ In one of the studies, nanoengineered granular hydrogel bioink was developed to enhance the mechanical properties of the structure and preserve the interconnected microporosity that helps in the cell attachment and tissue regeneration. The granular bioink also reduced the shear stress while printing using extrusion-based technique.^10^ In another study, a biphasic system consisting of solid and liquid phases were used to achieve the optimal properties while using extrusion based bioprinting technique. The bioink had solid colloidal particles suspended in liquid matrix creating a biphasic structure that provides structural support and flexibility by tailoring the viscoelastic properties of the ink and giving a control over the rheological behavior of the bioink during the extrusion.^11^ Jetting based technique comprises of inkjet and laser assisted bioprinting where inkjet based bioprinting utilizes droplet-based technique to deposit cell laden droplets that creates patterns. The requirement of this technique is using bioink having viscosity around 3–50 mPa.s, for smooth ejection of the droplets. This type of printing offers high resolution and can be used for fabricating small controlled structures.^12^ Laser assisted bioprinting transfer bioink droplet using laser pulses onto a substrate and requires bioink with moderate viscosity that can be vaporized using laser pulse. The printability using this technique is excellent with high resolution and spatial control. The velocity of the droplets that hit the substrate determines the shear force or the impact related stress. Cells may face damage such as cell death or altered cell behavior if the velocity is too high. By adjusting the jetting pressure and pulse frequency, the velocity of the droplet can be controlled. The size of droplet also plays an important role as the cell loading is higher in larger droplets but they can showcase higher impact force when they hit the surface.^13^ In one study, the researchers used polyvinylpyrrolidone as bioink additive that helped in enhancing the printability, making it more stable and viscous. It is important while using jetting-based technique for bioprinting to take care of the substrate surface as use hydrophilic surface encourages the droplet to spread out, leading to uneven deposition of cells and hydrophobic surface leads to droplet bead up which affects the cell adhesion to the structure.^14^ Another factor that effects jetting based bioprinting is the droplet evaporation, as it leads to bioink hardening which leads to cell death. To prevent droplet evaporation, bioprinting systems have controlled humidity chambers that reduce the rate of evaporation of solvent.^15^ Vat polymerization uses photopolymerizable resin or bioink that solidifies when come in contact to light. This technique includes stereolithography (SLA) and digital light processing (DLP). SLA uses laser beam that cures layer by layer and solidifies the resin selectively while offering high resolution and precision whereas DLP uses micromirror device that can project entire layer of light to the resin. It speeds up the printing process and can fabricate large complex organ scaffolds.^16^ It is important to balance the concentration of photoinitiator being used while printing as less concentration can lead to insufficient crosslinking and higher concentration can lead to cell toxicity.^17^ Instead of UV light in spectrum range 350–400 nm, it is better to use visible light (405–450 nm) that is biocompatible. The intensity of the light source use for curing is also important as higher intensity led to localized heat generation and lower intensities may cause prolong printing times and reduce resolution.^18^ All of these techniques are capable of delivering high precision during the construction of complicated biological structures. In extrusion- based technique, bioinks are It begins with the design using computer-aided design, whereby one develops a three-dimensional model of the intended structure of the tissue. Furthermore, a decision is made on a suitable bioink and its preparation for printing desired constructs through the collection of cellular and material components. The most significant factor in 3D bioprinting involves bioinks, living cells on a biomaterial base that offer an appropriate environment for cellular replication, adhesion, and differentiation; functional tissue constructs can eventually be developed.^19^ Such bioinks can be made from natural or synthetic material, their choice depending, of course, on the compatibility with the cells and/or special requirements of use. The selection of a bioink is mainly influenced by the type of bioprinter, printing technique, and required mechanical/biological properties.^20^ It is critical that bioinks, for optimal performance, have appropriate mechanical strengths, tunable gelation rates, biocompatibility, and be scalable for large-scale production.^21^ A number of characteristics need to be considered during formulation, including biocompatibility and biodegradability. The proposed biomaterial should have low immunogenicity and controllable biodegradation rate, matched by a tissue repair process.^22^ It is important that the bioink degrades itself, so it forms its own ECM.^23^ Besides, the viscosity of the bioink is important for an accurate deposition process: too high viscosity can block nozzles, and too low viscosity results in cell damage. Low viscosities can be further modified by chemical, physical, or enzymatic crosslinking of the bioinks.^24^ Water absorption and porosity are just a few of the factors involved in the mechanical integrity of the scaffold. Mainly, hydrophilic polymers are used because they can swell and still be able to maintain the shape of the scaffold. Moreover, the biomaterial to be used for such purposes should present biological interaction sites to integrate the cells to proliferate or adhere. In the absence of such sites, synthetic scaffolds can be functionalized with bioactive sequences, including RGD (arginine-glycine-aspartate) or MMP binding sites, to further enhance cellular interactions.^25^ Figure 2 summarizes the major considerations for the choice of bioinks, and a number of bioink types commonly applied in the context of tissue engineering applications are the subject of further discussion in the present review and the challenges faced while working with bioinks along with few innovative bioinks is shown in Table 1. The bioprinting techniques present significant challenges due to cell sensitivity which requires environmental factors such as nutritional supply, temperature, and pH. Additionally, there are risk factors that affect cell survival during the printing process, for instance, mechanical stress, viscosity, and shear-thinning, which are governed by bioink parameters. Currently, available bioinks have low printability which translates to fewer options in designing tissue constructs and, in a way, reduces cell functions because of post-printing operations like curing and incubation.^26^

**Table 1.** Challenges and properties of different types of bioinks.

Challenge	Aspect	Innovative solutions	Examples
Printability	Rheological properties	- Use of shear-thinning bioinks for smooth extrusion and rapid solidification. - Incorporation of nanoparticles to stabilize flow.	- Alginate-based bioinks. - GelMA reinforced with cellulose nanocrystals.
	Crosslinking strategies	- Dual-crosslinking (e.g. ionic and photo-crosslinking). - Thermo-responsive bioinks that gel at body temperature.	- GelMA-alginate crosslinked with calcium ions and UV light. - PNIPAAm for temperature sensitivity.
	Multimaterial systems	- Hybrid bioinks combining soft hydrogels and rigid materials. - Use of sacrificial materials for temporary support.	- Bioinks with PEGDA for vascular scaffolds. - Agarose as a sacrificial material for hollow tubes.
	Resolution and fidelity	- Bioinks that improve printing fidelity for complex geometries. - Use of modular bioink components like cell-laden microgels.	- Modular GelMA bioinks for creating organoids.
Cell viability	Biocompatibility	- Use of natural ECM-mimicking bioinks like collagen, fibrin, or hyaluronic acid. - Synthetic bioinks functionalized with bioactive peptides.	- Collagen-based bioinks for cartilage regeneration. - PEG functionalized with RGD peptides.
	Reducing shear stress	- Optimized nozzle designs to reduce extrusion force. - Use of low-viscosity bioinks for gentle cell encapsulation.	- Alginate bioinks extruded at low pressures to maintain cell viability.
	Cytoprotective measures	- Photoinitiators with low cytotoxicity for gentle UV crosslinking. - Antioxidants to mitigate oxidative stress during printing.	- Visible light photoinitiators like LAP (Lithium phenyl-2,4,6-trimethylbenzoylphosphinate).
	Homogeneous cell distribution	- Pre-encapsulation of cells in microgels. - Cell-friendly microfluidics for uniform distribution.	- Microgel carriers made of PEGDA for uniform encapsulation.
Innovative bioinks	Modular Bioinks	- Incorporation of pre-formed microspheres or cell-laden particles for structural control.	- Cell-laden GelMA microspheres for vascularized tissues.
	4D Bioinks	- Stimuli-responsive materials for dynamic behavior, like shape changes and stiffness modulation.	- PNIPAAm for temperature-sensitive scaffolds. - pH-responsive PEG-hydrogels.
	Multi-cellular Bioinks	- Integration of multiple cell types and biochemical gradients to recreate tissue complexity.	- Skin constructs with fibroblasts and keratinocytes in bioinks.

These issues are overcome by creating bioinks with good printability for next-generation bioprinting applications that do not sacrifice the proximity of the cell to the bioink. These advanced formulations aim to create ideal microenvironment for cells thus, speeding up the timeline for attachment, proliferation, and differentiation. Bioinks derived from natural polymers such as alginate, collagen, silk, and gelatin demonstrate promising results in terms of cellular activity and tissue functionality. Furthermore, enhanced mechanical properties of scaffolds improve the transport of nutrients and elimination of waste which also promotes growth or improves health of the cell populations. The additional supplementation of bioactive substances and growth factors further enhances cell activity and survival rate promoting increased regeneration processes.^28^

Studies conducted in vivo validate the effectiveness of bioprinted tissues in supporting regeneration when procedures are fine-tuned. These results highlight the importance of advancing bioink formulation and printing techniques to address current challenges and advance the clinical use of 3D constructs.^29^

## Bioinks

The various types of bioinks discussed are decellularized extracellular matrix, protein based, cell based and hybrid based bioink (Table 2) and the suitability with each bioprinting technique is discussed in Table 3. A new approach to personalized fabrications of tissues and organs is 3D bioprinting using decellularized ECM as a bioink. This highly advanced technique focuses on fabricating tissue-specific 3D constructs that accurately recapitulate the native tissues’ biochemical composition and mechanical properties. The idea of using dECM as a bioink is inspired by the fact that ECM contains critical proteins, which it binds, such as fibronectin, vitronectin, laminin, tenascin, collagen, and various proteoglycans, all important in aspects of tissue architecture and function.^30^ The general microenvironment of ECM is one of the most important factors in tissue survival, regulation of functions, and repair mechanisms because of their provided structural and biochemical cues to regulate cell behavior.^31^ When the ECM becomes remodeled or disorganized, these changes can promote various pathologies, which include cancer, hypertension, and osteoarthritis.^32^ A major benefit of using ECM as a bioink is that it allows cell recognition and attachment and, therefore, cell interaction with the ECM, without provoking immune responses. Decellularization is the process of removing the cellular components and nuclear material from the donor ECM with the avoidance of structural and biochemical integrity loss of the matrix.^33^ This ensures the natural biocompatibility of the resulting dECM, which may be an important factor in providing an advantageous microenvironment to the cells embedded within it for tissue regeneration. Since the decellularization process maintains the composition of the native ECM, this would allow the fabrication of scaffolds that provide for cellular attachment, proliferation, and differentiation-all important events in the development of functional tissue constructs.

**Table 2.** Different types of bioinks.

Type of bioink	Description	Advantages	Challenges	Application	Recent advances
Hydrogels	Water-swollen, crosslinked polymeric materials ideal for mimicking the extracellular matrix (ECM).	High water content, biocompatibility, and tunable mechanical properties.	Limited mechanical strength and slow degradation.	Tissue engineering, wound healing, and drug delivery systems.	Development of shear-thinning hydrogels for improved printability.
Decellularized Extracellular Matrices (dECM)	Bioinks derived from tissue ECM, offering a scaffold that mimics native tissue environments.	Natural biomimicry, supports cell attachment and tissue growth.	Complex extraction and potential for immune response.	Regenerative medicine, tissue-specific bioinks for organ printing.	Refined decellularization techniques to retain ECM bioactivity.
Nanomaterials	Nanoparticles and nanocomposites incorporated into bioinks to enhance mechanical properties and bioactivity.	Enhanced mechanical properties, potential for targeted delivery and bioactivity.	Toxicity concerns and challenges in uniform distribution.	Bone regeneration, drug delivery, and antimicrobial applications.	Integration of nanomaterials to enhance conductivity and bioactivity.
Cell aggregates	Tissue spheroids or cell clusters that self-assemble into functional tissue structures.	Self-assembling, promotes natural tissue development and maturation.	Difficult to handle and print due to aggregation behavior.	Tissue regeneration, organoids, and cell-based tissue models.	Advances in bioreactor technologies to support large-scale tissue maturation.
Synthetic polymers	Man-made polymers that can be tailored for specific mechanical and biological properties.	Customizable properties, controlled degradation rates, and mechanical strength.	Lack of biological cues and slower degradation.	Scaffold fabrication, bone and cartilage regeneration.	Innovations in synthetic biodegradable polymers with tunable degradation rates.
Natural polymers	Biologically derived polymers such as collagen, gelatin, and alginate, commonly used for their biocompatibility.	High biocompatibility, biodegradable, and supports cell growth.	Limited mechanical strength and inconsistent properties.	Soft tissue engineering, vascular tissue engineering.	Improved crosslinking methods for enhanced mechanical stability in natural polymers.
Composite bioinks	Combination of synthetic and natural materials to enhance structural and biological properties.	Combines the benefits of both synthetic and natural materials for tailored bioinks.	Complex formulation and optimization needed for printing.	Multi-functional tissues, hybrid scaffolds for complex tissue regeneration.	Hybrid bioinks combining nanomaterials and bioactive agents for complex tissue formation.

**Table 3.** Different bioink type and their suitability with 3D bioprinting techniques.

Bioink type	Extrusion bioprinting	Inkjet bioprinting	Laser-assisted bioprinting	Advantages
dECM	Highly compatible; optimal at 3%–8% (w/v).	Limited viscosity control; hybrid systems needed.	Rarely used; limited due to thermal sensitivity.	Tissue-specific biochemical cues; excellent for organoids and tissue scaffolds.
Protein-based	Versatile; GelMA optimal at 5%–20% (w/v).	Compatible at lower concentrations (⩽10% w/v).	Collagen effective for high-precision constructs.	Biocompatible, biodegradable, and tunable for various tissue types.
Cell-Laden	Ideal for direct cell deposition; supports high densities.	Limited by cell settling and nozzle clogging.	Rarely used; precision is challenging.	Enables heterogeneous tissue creation and high cell viability.
Hybrid (composites)	Excellent for robust scaffolds; hybrid bioinks perform well.	Limited to low-viscosity hybrids.	Emerging use in combining precision and stability.	Combines mechanical strength with bioactivity, useful for bone and load-bearing tissues.

### Decellularized extracellular matrices

**Figure 3.**
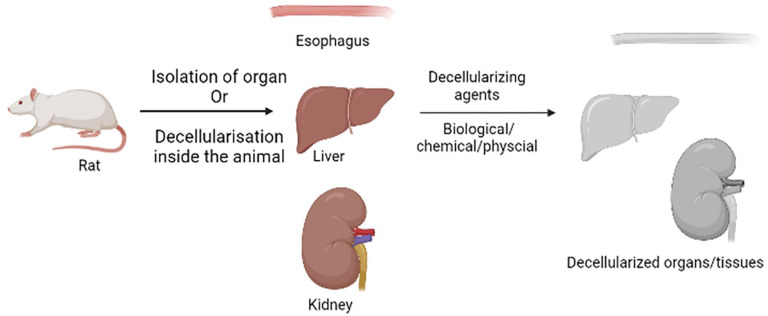
Decellularization of organs.

Different decellularization approaches, including chemical, enzymatic, and physical decellularization techniques, are being widely performed in an effort to decellularize tissues and organs of both animal and human origin for transplantation purposes. Each technique should remove cells/cellular components without compromising the three-dimensional structure of the ECM (Figure 3). The majority of the chemical methods involve acid-base treatments, chelating agents, detergents such as SDS, and organic solvents that disrupt the cell membrane and make the cell materials soluble. The enzymatic decellularization in most cases involves enzymes such as trypsin and nucleases, which can depose proteins and nucleic acids by degrading them and thus ultimately allow the removal of cellular components. These are normally done through physical methods such as freeze-thaw cycles, mechanical forces, and agitation in such a way that the damage to the ECM is minimal. These agents, in certain specific decellularization protocols, are used in combination for the efficient decellularization of tissues and organs. Examples include two-step perfusion decellularization, whereby in this method, chemical or enzymatic agents are delivered via the vascular system to target cells throughout the organ (Table 4). Decellularization techniques alone or in combination can remove donor cells from the tissue and preserve ECM proteins along with the signaling molecules that are entrapped in ECM.^34^ Taylor et al., decellularized the whole body of adult Sprague-Dawley rats using a perfusion technique. In such an approach, 1% SDS was perfused through the vasculature via a small incision in the inferior vena cava. Once the organs became translucent during the process of decellularization, the vascular connections were then ligated and further continued until skeletal muscles became translucent. It was also a straightforward, yet versatile methodology with a great potential of translation into biologically relevant whole organ scaffolds, due to minimal disruption to the natural structure of the ECM.^35^ Another sophisticated method includes the pressure gradient, where decellularizing agents are pushed through tissues under controlled pressure, thus displacing efficiently cellular debris while maintaining ECM integrity. By contrast, supercritical fluid decellularization of tissues refers to the use of inert substances with lower viscosity, like supercritical CO₂, which removes cells while causing minimal alteration in the structural and biochemical properties of the ECM. Agitation and immersion involve submerging tissues in decellularizing solutions with mechanical agitation to improve the penetration of decellularizing agents.

**Table 4.** Various decellularizing agents for production of decellularized extracellular matrix.

Decellularizing agent	Sub-classification	Examples	Technique/Process	References
Chemical agents	Chelating	EDTA, EGTA	Binds to divalent cations, disrupting the cell-ECM adhesion	Akbari Zahmati et al.^36^
	Acid-base	Acetic acid, NaOH	Solubilizing the cytoplasmic components	Choi et al.^37^
	Detergents	SDS, Triton X-100, CHAPS	Disrupt and solubilize lipid-lipid interactions and nuclear components respectively.	Crapo et al.^38^
	Solvents	Alcohols, Acetones	Dehydrates, solubilize and removes lipids	Keane et al.^39^
Biological agents	Enzymes	Trypsin, Nucleases	Peptide bond cleavage, catalyze hydrolysis of nucleic chains	Khan et al.^40^
Physical agents	Freeze-thaw	Varied temperature	Disrupt cell membrane	Choi et al.^37^
	PEG	Different molecular weights	Destabilization	Uchimura et al.^41^
	Mechanical agitation		Cellular disruption	Ota et al.^42^
	Mechanical force	Hydrostatic pressure	Cellular disruption	Shafiq et al.^43^

EDTA: ethylenediaminetetraacetic acid; EGTA: ethylene glycol tetraacetic acid; NaOH: sodium hydroxide; SDS: sodium dodecyl sulfate; CHAPS: 3- [ (3-cholamidopropyl) dimethyl ammonio]-1-propanesulfonate; PEG: polyethylene glycol.

All these factors, and more, including tissue thickness, type of decellularizing agent, and strength of agitations, condition the duration of the process.

For decellularization, original sources of ECM span a wide range, from animals and humans to even plants. Pati et al. successfully obtained dECM from porcine cartilage and heart tissue that was further used in 3D bioprinting of cell-laden hydrogels.^44^ These hydrogels exhibited enduring function in supporting high cell viability and survival for extended periods of time.^45^ In another work, Toprakhisar et al. fabricated dECM hydrogel from bovine Achilles tendon by adopting the microcapillary-based technique of bioprinting. Cell viability studies preformed on these hydrogels demonstrated no cytotoxic effects on mouse fibroblasts, which confirmed their biocompatibility.^46^ Plant ECM scaffolds have also been used, with works like that of Fontana et al., who performed decellularization on plant tissues of species such as Calathea zebrina, Anthurium waroquaenum, vanilla, and Laelia angustifolia. These decellularized plant scaffolds subsequently had adaptable platforms for tissue engineering applications.^47^ Gerli et al. demarcated the possibility of producing a decellularized composite tissue scaffold starting with an intact cadaveric human upper extremity, thusertilizing toward a future where whole-human tissue scaffolds can be applied in regenerative medicine.^48^ Campinoti et al., combined ECM-scaffold technology using human amnion epithelial cells and ECM-adhesion proteins to evaluate differentiation of epithelial cells into hepatocyte-like cells and understanding the effects of it. They used 4D bioreactor to provide oxygen and media continuously and it was observed that cells differentiated showed hepatic positive markers and secreted albumin and urea. The innovative ECM-scaffold technology can support differentiation of cells and can be used for fabricating bioengineered constructs for transplantation.^49^

#### Method of isolation

##### Formulation of decellularized bioink

The formulation of bioink for 3D bioprinting using decellularized extracellular matrix (dECM) involves three critical steps: tissue processing, decellularization, and post-decellularization treatment.

*a) Tissue Processing—*Initial preparatory steps involve removal of the large anatomical structures from the tissue, such as fat deposits and major blood vessels. Following cleaning, the tissue is then cut into little, more workable pieces and washed extensively with either water or PBS. This washing step is an important concern not only in removing any debris from the tissue but also for a pre-treatment condition in subsequent chemical or enzymatic treatment.
*b) Tissue Decellularization—*Then, decellularization is performed using a decellularizing agent that depends on the properties of the tissue and its composition of ECM, cell density, and overall structure. The most commonly used decellularizing agents are detergents, including SDS, enzymes such as trypsin, acids, and bases. Except for the nuclear material, the removal of all cellular components without compromising the structural integrity and biochemical composition of the native ECM is the primary goal. In this respect, the decellularization methods to be employed will have to be selected with care and optimized-the compromise between effective cell removal with minimum damage to the architecture of the ECM, which is key to retaining its bioactivity and mechanical properties for tissue regeneration.
*c) Post Decellularization Treatment—*Decellularization is followed by the removal of residual chemicals or decellularizing agents, which is important to avoid cytotoxic effects on later-populating cells within the bioink. Indeed, this often occurs via multiple rounds of washing with solutions such as PBS. Then, the tissue is sterilized in general to be free from microbial contaminants by agents such as peracetic acid or ethanol. Lyophilization follows sterilization, during which the residual moisture is removed from the tissue and the tissue is ground into a fine powder. This powdered decellularized tissue form represents one of the critical intermediates in the bioink formulation process.

After the powder is obtained from lyophilization, it undergoes enzymatic solubilization with enzymes such as pepsin and papain, which eventually degrade the ECM components into liquid. The subsequent liquid form of dECM then serves as the base material for bioink in 3D bioprinting applications. The solubilized dECM retains the bioactive properties of the native ECM, thus supporting cell adhesion, proliferation, and differentiation in tissue-engineered constructs. This will be enabled by controlling every step, from the synthesis of the building blocks to the manipulation of the curing conditions of the resultant constructs, to develop functional bioinks with biomimetic features similar to those of natural tissue environments and support the fabrication of biologically relevant 3D structures for applications in regenerative medicine.

##### Types of crosslinking for dECM bioinks

Various crosslinking methods are still continuously being applied to dECM bioinks to improve their cell proliferation and adhesion, among other properties that enhance overall stability within the 3D-printed construct. These crosslinking methods are important in improving structural integrity and biological functionality during and after printing.

In a work by Ahn et al., it was presented that the development of the 3D cell printing system is with a heating modulus. Because of this heating modulus, precise stacking of the dECM-based constructs was allowed in this work. This modulus, during printing, can allow simultaneous heat crosslinking of the bioink, hence making the correct formation and stabilization of its layered structure possible.^50^ Complex 3D structures can be assembled by this method with more remarkable mechanical properties compared to simple mixing.

Kim et al. presented a dECM bioink containing hTMSCs and dECM, emulating the biochemical composition of native cornea. This bioink showed excellent 3D shape stability through thermal crosslinking and high cellular viability.^51^ Besides, hTMSCs in the bioink exhibited lineage-specific expression in keratinocytes, a characteristic required for corneal tissue engineering. The essential development of functional biomaterials in regenerative medicine is basically forming bioinks that can mimic the properties of native tissues.

Jang et al. developed a two-step crosslinking strategy to prepare the heart tissue construct with good mechanical strength and structural stability. Vitamin B2 was used as a photoinitiator for the crosslinking of pepsin-digested dECM solutions through layer-by-layer photocrosslinking using UV light. Each layer was then thermally crosslinked to further stabilize the construct. Consequently, 3D constructs with native cardiac tissue mechanical properties were achieved, as well as other enhanced cardiomyogenic differentiation factors, which are an important factor for cardiac tissue engineering applications.^52^ Such dual-crosslinking strategy provided mechanical integrity while maintaining biological function.

In another study, bio-artificial blood vessels were prepared by Gao et al. through ionic and thermal crosslinking methods. Alginate was mixed with vascular tissue-derived dECM and coaxially printed with the CPF-127 solution. During printing, alginate underwent ionic crosslinking with CaCl₂ contact, enabling initial shape fidelity and subsequently forming tubular constructs. Finally, after printing the construct, incubation in a 37°C atmosphere allowed the thermal crosslinking of it; subsequent removal of the CPF-127 solution revealed the bio-blood vessel.^53^

When implanted in vivo, the fabricated vascular tissues demonstrated significantly improved recovery from ischemic injury, which supports the possibility of crosslinked dECM bioinks being used as a therapy in vascular tissue engineering.^53^ These examples give an idea of how different strategies of crosslinking-thermal, ionic, and photo-crosslinking-play an important role in developing dECM bioinks with improved mechanical properties and biological performances. The capability to fine-tune these crosslinking processes is considered crucial for the development of tissue-specific scaffolds that more accurately mimic both the function and architecture of the native tissue.

#### Applications of dECM bioinks

Decellularized bioinks have enormous interest in the development of highly biomimetic native tissues. Mao et al. fabricated a microtissue with cell-laden bioinks from decellularized porcine liver extracellular matrix (dECM). dECM was mixed with GelMA and hiHep to achieve appropriate bioink for 3D printing of a microtissue. A DLP-based printer has been used to print this microtissue. This augmented the printing capability of the bioink from the inclusion of dECM and improved cell viability. This liver-specific microtissue holds great promise in liver tissue engineering applications as they show good biocompatibility and promote HepG2 and stem cells development and proliferation.^54,55^ In a comparative study, Jeong et al. explored the effect of different detergents on the quality of liver dECM. The decellularized agents to be tested included SDS, SDC, Triton X-100 (TX), and a combination of Triton X-100 with ammonium hydroxide (TXA). Following characterization assays in the study, TXA was found to be the best detergent for liver dECM bioink preparation due to the fact that it provided enhanced printability, while maintaining maximum ECM content and ensuring greater cell compatibility with optimal gelation time.^56^ Zhang et al. prepared a cartilage tissue engineering bioink with silk fibroin, dECM, and bone marrow mesenchymal stem cells. The 3D printed constructs manufactured with this bioink had an accurate outer morphography, an appropriate degradation rate, and the printed constructs promoted the proliferation of BMSCs. Moreover, chondrogenic differentiation was induced, thus it might be a very promising strategy for the regeneration of cartilage.^57^

Cell-derived matrices (CDM) share similarities with decellularized extracellular matrices (dECM), though they differ in the protein sources. Unlike dECM, where proteins are derived from actual animals, CDM is produced through in vitro processes. This method offers advantages such as customizable composition based on specific applications, consistency in production, reduced reliance on animal use, and lower immunogenicity. However, a challenge with CDM is the loss of the native tissue’s physical properties, even though the biochemical composition remains largely intact.^58^

The fabrication of CDM can be done either through 2D or 3D cell culture depending on the required format of the product. Generally, the steps of fabrication involve the following: cell expansion, seeding, matrix generation, and decellularization. Cell isolation from the source, characterization, expansion, and cryopreservation of cells are done during the cell expansion phase for future use.^59^ Then, cryopreserved cells are thawed and cultured; passaging is repeated until an appropriate quantity of cells is achieved. During cell expansion, maintenance of healthy cell morphology and the preservation of stemness are important, since these greatly affect ECM production. Upon confluence, cells are then seeded onto either 2D surfaces or 3D scaffolds depending on experimental/clinical needs.

The process of matrix generation involves the creation of optimal conditions that favor the derivation of the ECM. The resultant ECM consists of a highly organized biomolecular and cell signaling factor network.^60,61^ The last step is decellularization; this involves the removal of cellular components using physical, chemical, or enzymatic treatments without compromising the structural and biochemical integrity of the developed ECM. This step has been identified to play a key role in minimizing the immunogenicity of the CDM material.

Since CDM is generated entirely in vitro using animal-origin stem cells, its properties and composition can be significantly altered by modifying various factors. For instance, increasing collagen content can be achieved by incorporating ascorbic acid, growth factors, and matrix proteinase inhibitors.^62,63^ Synthetic macromolecular agents like Ficoll^®^ and Polyethylene Glycol (PEG) can also promote better matrix deposition and alignment.^64^ Adjusting cell culture conditions can enhance collagen production by fibroblasts through factors like hypoxia, substrate stiffness influencing ECM composition and properties, and substrate topography affecting structural organization. The concept of macromolecular crowding (MMC) was introduced to increase the medium’s density, which improves viscosity and reduces diffusivity. MMC surrounds cells with macromolecules, facilitating the movement of components by excluding the medium and causing steric hindrance.^65^ MMC regulates ECM by altering cell behavior, ECM expression, and the cell medium environment.^66^ It also enhances enzyme activity, promotes the expression of C-propeptide-supporting proteins, and stabilizes enzyme-substrate interactions.^67^ Hydrogels formed under MMC conditions have been observed to exhibit increased mechanical stiffness.^68^ Macromolecular agents like bovine serum albumin, PEG, polystyrene, dextran, and Ficoll^®^ enhance CDM production when combined with MMC.^69,70^ CDMs are subject to extensive characterizations, similar to other biomaterials, including physical, chemical, and mechanical, and in vitro and in vivo validations.^71,72^ The biochemical analyses generally entail the quantification of collagen, sGAGs, and DNA. These are typically quantified using hydroxyproline assays, assays for GAG, and DNA quantification kits, respectively. Generally, proteomic analysis for the type of proteins present in CDMs is normally done through mass spectrometry, which helps in ascertaining specific markers of stem cells and quality control.^73^ Scanning electron microscopy is performed to observe the surface structure of CDMs, while transmission electron microscopy is conducted to avail the assessment of collagen fiber arrangement.^74^ Atomic force microscopy provides force-distance curves that give insight into nanoscale mechanical properties.^75^ In testing the removal of cellular components, fluorescent dyes such as DAPI and Hoechst are used to stain DNA, which allows the remaining nuclei to be observed with microscopy.^76^ Alexa Fluor dyes and DiI (1,1′-dioctadecyl-3,3,3′,3′-tetramethylindocarbocyanine perchlorate) are used to stain F-actin and cell membranes, respectively, for resolution imaging of cellular components.^77^ Alcian blue normally serves for the analysis of proteoglycan deposition, whereas Picrosirius red staining is employed for the detection and hence for ascertaining the distribution of collagen fibers.^78^ For protein profile estimation, SDS-PAGE is employed, while the identification of specific proteins is carried out by Western blotting.^79^ These are the various characterization techniques normally utilized in providing complete details about CDMs, making them potential substrates for fabrication into tissue-engineered constructs. The most widely investigated function of CDMs relates to the regeneration of cartilage, and several studies have utilized chondrocytes, synovium-derived stem cells, and BMSCs. In one such study, porcine chondrocytes were used by authors to fabricate CDMs for cartilage regeneration and reported improved cartilage regeneration upon the use of a multi-layered membrane structure in conjunction with bone marrow stimulation therapy in a canine model. Thus, the decellularized CDM membrane accelerated cartilage tissue formation in place.^80^ In yet another example, osteogenic differentiation in mesenchymal stromal cells seeded on titanium fiber mesh scaffolds is induced by using rat-derived CDMs and thus could have possible applicative uses in bone tissue engineering.^81^ Lu et al. fabricated CDM scaffolds with human BMSCs, human articular chondrocytes, and normal human dermal fibroblasts. The scaffolds used a knitted poly(lactic-co-glycolic acid) PLGA mesh as a sacrificial template and were then implanted in mice. Results showed excellent biocompatibility with a very low host immune response, proving the potential of such CDMs for repair of cartilage.^82^ Given the severe shortage of cadaveric allogenic tissues and the risk of cross-contamination from xenogenic sources,^80,82–85^ CDMs also hold promise in cardiovascular tissue engineering. Wystrychowski et al. report the first clinical application of CDMs for blood vessel regeneration using fibroblast-derived vessels provided by Cytograft Tissue Engineering. Grafts were stored at −80°C and were devitalized, containing only fibroblasts and no endothelial cells, and were successful in the clinical regeneration of human blood vessels^86^ Apart from tissue engineering, CDMs find their increasing applications in disease modeling, understanding disease mechanisms, identification of potential therapeutic targets, and personalized treatment development.^87^ On one such instance, endothelial CDMs were studied for their potential roles as promoters of hepatocyte-like cells in liver disease models. Such models were studied for the interaction of the hepatocytes to the non-parenchymal CDMs, specifically how the signals of the ECM might influence transcriptional and nuclear factors that exist.^88^ Moreover, the future of CDMs looks very promising in neurodegenerative disease studies because they have the potential to modulate neural cell adhesion and proliferation. It has been indicated that CDMs support a complex cellular system comprising neurons, astrocytes, and oligodendrocytes for potential applications in the treatment of brain lesions and as disease models.^89^

### Protein based inks

**Figure 4.**
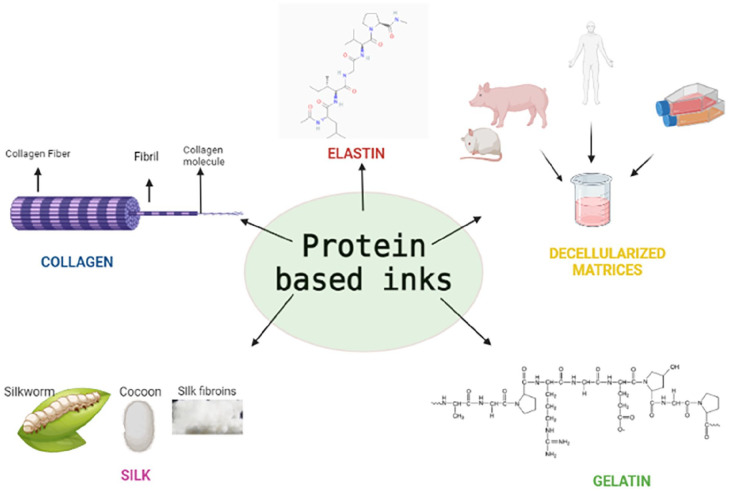
Different types of protein based bioink.

Naturally occurring proteins like collagen, gelatin, keratin, silk, and elastin belong to one of the most abundant proteins available (Table 5 and Figure 4). Many of these proteins possess favorable properties, including biocompatibility, biodegradability, tunable physiochemical, biological, and mechanical properties. The advantages from these properties make this class of proteins promising biomaterials for applications in 3D bioprinting. Compared to synthetic polymers, biologically derived proteins offer advantages such as reduced immunogenicity, better degradability, and better biocompatibility-these are the main reasons for choosing such materials to fabricate complex hierarchical structures. The addition of proteins optimizes viscosity, enhances printability, and shape fidelity in bioprinting applications. These bio-polymers are biodegradable as well as renewable, which reduces dependency on synthetic polymers derived from fossil sources.^90^ In the following section, protein-based bioinks are discussed in detail.

**Table 5.** Classification of protein based bioinks.

Protein-based ink	Sub-classification	Examples	Technique/Process	Limitation	Application
Collagen-based bioinks	Natural proteins	Type I, Type III Collagen	Extrusion-based printing, Inkjet bioprinting	Low mechanical strength, difficult to crosslink.	Skin tissue engineering, bone regeneration.
Fibrin-based bioinks	Natural proteins	Fibrin gel, Fibrinogen	Extrusion-based printing, Laser-assisted bioprinting	Rapid degradation, limited mechanical properties.	Wound healing, vascular tissue engineering.
Silk fibroin-based inks	Fibrous proteins	Silk fibroin	Electrospinning, Extrusion-based printing	Limited availability, complex processing.	Tendon, ligament regeneration, skin scaffolds.
Elastin-based bioinks	Structural proteins	Tropoelastin	Inkjet printing, Extrusion-based printing	Difficult to handle, slow degradation rates.	Vascular tissue engineering, wound healing.
Keratin-based bioinks	Fibrous proteins	Keratin hydrogels	Extrusion-based printing, Electrospinning	Limited mechanical strength, variability in properties.	Wound healing, bone tissue engineering.
Gelatin-based bioinks	Hydrolyzed collagen	Gelatin-methacryloyl (GelMA)	Photopolymerization, Extrusion-based bioprinting	Thermal sensitivity, low mechanical stability.	Soft tissue engineering, cartilage repair.
Albumin-based bioinks	Serum proteins	Bovine serum albumin (BSA)	Extrusion-based printing, Crosslinking with hydrogels	Poor mechanical properties, challenging to print.	Drug delivery, soft tissue repair.
Matrigel-based bioinks	ECM protein mixtures	Matrigel (Basement membrane matrix)	Inkjet printing, Extrusion-based bioprinting	Expensive, variability in batch composition.	Organ-on-chip models, cancer research.
Laminin-based bioinks	ECM proteins	Laminin-511, Laminin-111	Coaxial extrusion, Inkjet bioprinting	Complex to isolate, limited long-term stability.	Nerve regeneration, tissue scaffolds.
Fibronectin-based bioinks	ECM proteins	Recombinant fibronectin	Extrusion-based printing, Electrospinning	Difficult to work with, poor mechanical properties.	Cell adhesion, wound healing.

#### Collagen

Collagen represents the most abundant mammalian protein, comprising approximately 30% of the total protein mass. It generally originates from porcine, bovine, murine, and marine sources.^91^ As one of the critical components of the ECM, collagen is part of the family of fibrous glycoproteins. By nature, collagen is hydrophilic, consisting of three polypeptide chains, also referred to as alpha chains, which twist in a triple helical manner. According to the combination of some of these helical domains, there are about 28 different types of collagen. The best type, collagen type I, is also the most abundant and is present in skin, tendons, and bones. It belongs to the category called fibril-forming collagens, wherein its triple helix structure is made of three alpha helices. Collagen type II is mainly present in cartilage.^92^ This so-called “repetitive glycine motif,” where in effect every third amino acid is a glycine, accounts for the triple-helical arrangement of collagen. Because of its excellent biocompatibility and low immunogenicity, collagen has recently become popular in tissue engineering, especially in the biomaterials used for 3D printing or scaffold fabrication. Physiologically, at a pH of neutrality and a temperature of 37°C, collagen molecules self-assemble into fibrils, forming a hydrogel. Such a property makes collagen the ideal material to develop scaffolds in tissue engineering applications.

##### Isolation of collagen

This extraction of collagen varies with the source and kind of tissue. In addition, the solubilization of collagen is not that easy due to the fact that its structure is stabilized through intermolecular interactions and crosslinking. Proper pretreatment before hydrolysis is very important so as to remove the non-collagenous substances that result in impurities in the isolated collagen.^93^ Collagen can be isolated by both the chemical and enzymatic hydrolysis method. Generally, at low pH, acids solubilize collagen fibers through chemical hydrolysis. Acetic acid usually gives better results to break the inter-chain crosslinkages of uncrosslinked collagen.^94,95^ On the other hand, enzymatic hydrolysis is employed by using enzymes like trypsin, chymotrypsin, pepsin, alcalase, and papain. This method allows more control over the degree of hydrolysis and specificity. Enzymes used at low concentrations cleave amino acids and remove carboxyl-terminal telopeptides that cause depolymerization of collagen. The product will be of value in reducing immunogenic and allergic reactions.^96^ In a study by Koch et al., a 3D printed construct of skin tissue was fabricated using fibroblasts and keratinocytes embedded in collagen.^97^ The laser-assisted bioprinting technique was used in order to arrange the cells in 3D patterns. Coating the surface with 20 layers of fibroblasts was done, to which 20 layers of keratinocytes were added within the collagen hydrogel.^98^ This construct for the generation of dermis and epidermis was further cultured for 10 days, checking cell activity and viability, which increased cell proliferation and adhesion accordingly.

##### Formulation of bioink

To optimize printability and prevent premature crosslinking, collagen is kept on ice during preparation, while the cellular components are maintained at 37°C to protect the cells from stress.^99^ For a uniform bioink, the collagen and cell suspension are combined and cooled, after which the hydrogels are prepared in syringes and used for 3D printing.^100^ The predominant methods for collagen bioprinting include micro-extrusion, inkjet, and laser-assisted printing techniques.

##### Crosslinking of collagen bioinks

**Figure 5.**
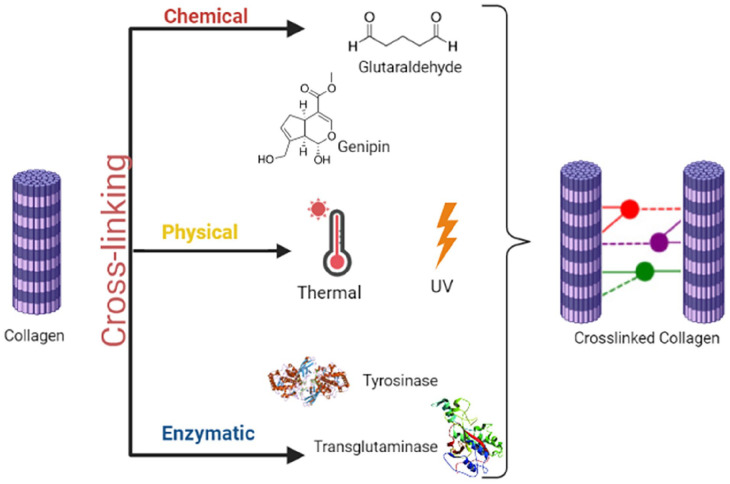
Crosslinking of collagen.

Crosslinking of collagen bioinks is unavoidable because after extraction, the native state crosslinks or their assembly structure may be disrupted. Such events may lead to the loss of its thermal stability, mechanical properties, and resistance to hydrolytic or enzymatic digestion. The method of crosslinking selected may have impacts on the microstructure, diffusive properties, and mechanical behavior of the bioink.^101^ Rarely, there exist three major forms: crosslinking by chemical, physical, and enzymatic means Figure 5.

While chemical crosslinking, collagen forms crosslinks between collagen monomers by reacting with a chemical agent via its carboxylic and amino functional groups. Glutaraldehyde is used commonly as a physical crosslinking agent because of low cost, high solubility, and wide availability. For example, Harriger et al. used glutaraldehyde for the fixation of collagen-based implants for the treatment of burn wounds. N-hydroxysuccinimide is another chemical agent, which acts similarly to target carboxylic groups in mediating bond formations between the aspartic and glutamic acids.^102^ Genipin is a biocompatible crosslinker product obtained from the iridoid glucoside in Gardenia jasminoides to form monomeric or oligomeric bonds with free amide groups in lysine, arginine, and collagen.^103^

This enzymatic crosslinking method offers enzymes like tyrosinase, laccase, and transglutaminase, capable of providing crosslinks in protein-based substrates.^104,105^ This approach has been developed to solve problems associated with toxicity and biological incompatibility of some chemical crosslinking agents.^106^

For this, physical crosslinking methods such as heating, drying, and irradiation have been employed in order to avoid the introduction of agents that may be harmful. In this process, the removal of water molecules in thermal crosslinking results in the subsequent formation of amide bonds between collagen molecules. Another advantage within UV crosslinking is the efficiency through which free radicals in aromatic amino acids are allowed to generate reactions with other molecules at faster rates.

#### Keratin

Keratin is a widely available protein found in human hair, wool, nails, hooves, and horns, composed of various amino acids such as cystine, glycine, proline, and serine, with low levels of lysine, histidine, and methionine. Due to processing challenges, there has been limited exploration of keratin in tissue engineering. However, keratin is an affordable material known for its high stability, low solubility, and ability to self-assemble into hydrogels.^107^ It contains two key cell-binding motifs, leucine-aspartic acid-valine (LDV) and RGD, which facilitate cell attachment. These motifs also eliminate the need for additional post-processing to include binding sequences, simplifying the manufacturing process.^108,109^ Keratin from wool and epidermal tissue contain 4%–8% and 2% sulfur, respectively, classifying them as hard and soft keratin.^110^ A skin substitute was developed using electrospun keratin and polycaprolactone (PCL), with keratinocytes and fibroblasts cultured to mimic native skin tissue. To prevent folding and tearing, a support layer of PCL was printed between fibroblast microfibers and keratinocyte nanofibers. When tested as a skin substitute for wound healing in animal models, the cell-seeded keratin-PCL scaffold showed superior healing compared to the PCL scaffold alone.^111^

##### Method of isolation

Isolation of keratin can be done using the following methods: oxidation,^112^ reduction,^113^ alkaline extraction,^114^ and use of ionic liquids.^115^

**Reduction method***—*In this method, targeted specific reagents such as mercaptoethanol, thioglycolic acid, and thiourea break the disulfide bonds in keratin. For example, at different pH conditions, mercaptoethanol reduces cystine disulfide bonds into cysteine. Yet, this method is burdened with several disadvantages such as high cost, toxicity, and harmful nature of the reducing agents. On the other hand, extraction by alkali relies on the depolymerization of the principal keratin chain as a result of the hydrolysis induced by alkali, which is neutralized by using acids. Ionic liquids, considered friendly to the environment, result from the combination of salts with organic cations and organic or inorganic anions. Under a nitrogen atmosphere, extraction by the ILs is reportedly able to take place from some substrates such as wool. Keratin is hydrophobic, and since ILs are highly polar, they disrupt hydrogen bonding, thus making ILs very useful in its isolation. The types of ILs include 1-allyl-3-methylimidazolium chloride ([Amin]Cl), 1-butyl-3-methylimidazolium chloride ([Bmim]Cl), and 1-butyl-3-methylimidazolium bromide ([Bmim]Br). Different oxidation methods, using agents such as peracetic acid and formic acid, have been in use since the early days for the isolation of keratin and are reported in literature.^116^ It deals with the cleavage of disulfide bonds, reorganizing into sulfonic acids. It is well accepted that the oxidation method requires a lot of time.

##### Formulation of keratin solution

Keratin is usually extracted by dissolving wool in a solution of urea/sodium dodecyl sulfate/mercaptoethanol. After that, it is heated up to a certain temperature, followed by stirring and filtration. The filtrate obtained undergoes dialysis and is then stored at 4°C in a keratin solution. Ethylene glycol diglycidyl ether or glycerol diglycidyl ether can be utilized for crosslinking in order to impart additional properties to the material.^117^

Keratin extraction from human hair is done with the use of peracetic acid and tris base. It is subsequently sieved and centrifuged to obtain a solution. This solution is subjected to filtration and dialysis to yield a purified solution of crude protein, which is lyophilized. Bioink preparation: The lyophilized keratin is mixed with PBS and then combined with a photosensitive initiator-catalyst-inhibitor solution comprising riboflavin, sodium persulfate (SPS), and hydroquinone.^118^

#### Silk

Silk is one of the most abundantly available naturally derived polymers and has been closely scrutinized for its potential in applications regarding tissue engineering and biomedical functions due to its unique properties.^119^ Further known for its lustrous appearance, dyeability, and breathability, silk became a popular material in the textile industry. However, its excellent mechanical strength, biocompatibility, flexibility, and hierarchical structure have also attracted remarkable attention from researchers. It is also produced by a number of arthropods, including spiders, crickets, bees, silkworms, fleas, and glowworms to build nests, cocoons, and webs.^120,121^ The life cycle of Bombyx mori silkworms includes three stages in life: laying eggs, hatching larvae, and spinning fibers into cocoon fiber. The silk fiber is obtained by sacrificing a silkworm through its middle metamorphosis stage. The two major proteins that make up silk fibers are fibroin and sericin. Fibroin is the inner structural element within silk that develops its mechanical property, while sericin refers to a glue-like coating around fibroin. A silk fiber consists of two filaments of SF, which in turn are constituted of connected nanofibrils.^122^ These nanofibrils are closely packed to form microfibrils, and along with the parallel arrangement of fibroin, they contribute to the exceptional mechanical properties of silk.^123^ The fibroin protein is a polypeptide with a molecular weight ranging from 200 to 350 kDa, comprising repetitive hydrophobic heavy chains and hydrophilic light chains that are linked through disulfide bonds. Major amino acids constituting the primary structure of the silk fibroin include glycine, serine, and alanine. The hierarchy of silk structures, along with spinning conditions and amino acid sequence, determines the biological and physical properties of various types of silk fibroin.^124^ Silk is lightweight with higher tensile strength than other biomaterials, such as collagen and PLA. Due to this, silk becomes an attractive option to carry out a variety of biomedical functions.

##### Processing of SF biomaterials

Generally, sericin needs to be removed by degumming for processes using raw cocoons. For instance, cocoons are boiled in a sodium carbonate solution, followed by rinsing with distilled water and drying to obtain the treated fibroin filaments.^125,126^ In this process, the sodium carbonate concentration and boiling temperature should be kept to prevent cleavage of the disulfide bonds. The dissolution of silk fibroin was done in lithium bromide, Agisawa’s reagent, lithium thiocyanate aqueous solution, and hexafluoroisopropanol (HFIP), all of them at the correspondent specific parameter that can affect the SF solubility.^127^ After that, the SF is submitted to a dialysis against pure water in order to remove excess of electrolytes. The resulting fibroin is stored at 4°C. Depending on the form of the material, further processing is carried out, such as making films of SF or depositing the fibroin fibers on a plate and then allowing to dry overnight.

##### Crosslinking of SF

Hydrogels can be used to crosslink SF through the use of various crosslinking methods. In physical crosslinking, proteins self-assemble through hydrophobic interactions on a normal basis; this takes time. Acceleration can be attained by altering parameters such as pH, temperature, vortexing, or using dehydrating agents. Chemical crosslinking utilizes agents such as hydrogen peroxide and horseradish peroxidase to initiate chemical reactions that functionalize the silk.

##### Applications of SF-base materials in biomedical field

SF was considered one of the finest scaffold biomaterials that possessed biocompatibility, non-immunogenicity, mechanical strength, and resemblance to native extracellular matrices for biomedical applications. A number of attempts have been made to design scaffolds combining SF with HA for bone tissue engineering purposes. By nature, such a scaffold very closely resembles natural bone; while HA constitutes the brittle osteogenic bioceramic part, silk forms the elastic and flexible portion. These hybrid systems can enhance both osteogenesis and angiogenesis. The study concluded that this system may be used in various forms such as films, hydrogels, and 3D printed scaffolds.^128^ In related studies, Sun et al. fabricated 3D scaffolds with graduated pore sizes using this SF-HA system. To further investigate the scaffold’s osteogenic capacity in the healing of bone damage, BMP-2, a potent osteoinductive cytokine, was delivered to SF scaffolds. Results obtained showed that BMP-2-loaded SF scaffolds remarkably enhanced the bone volume and mechanical strength, hence proving to be a potential treatment for acute bone injuries in the absence of hMSCs.^129^

### Hybrid based inks

One biomaterial cannot meet all the responsibilities of bioinks and fabricate biomimetic tissue-like constructs. To address this issue, many studies have been focused on hybrid or composite biomaterials for enhancing the performance of bioinks through different components (Table 6). The biomaterials can be categorized into several classes based on their composition and functionality.

**Table 6.** Characteristics of different hybrid bioinks.

Type of bioink	Material	Examples	Technique/Process	Limitation	Application	Recent Advances
Bioinks with natural biomaterials	Collagen	Collagen type I	Extrusion-based printing, crosslinking with UV or thermal processes	Weak mechanical properties	Tissue engineering, wound healing, organ printing	Development of collagen-based bioinks for 3D printing vascular networks
Bioinks with natural biomaterials	Alginate	Alginate hydrogels	Ionotropic gelation with calcium ions	Poor cell adhesion	Drug delivery, wound dressings, cartilage tissue engineering	3D bioprinting for complex tissue structures using alginate blends
Bioinks with natural biomaterials	Gelatin	Gelatin Methacryloyl (GelMA)	UV crosslinking, extrusion-based 3D printing	Rapid degradation	Soft tissue engineering, 3D bioprinting, wound healing	Development of photo-crosslinkable GelMA bioinks for higher resolution
Bioinks with natural biomaterials	Fibrin	Fibrin hydrogels, Fibrinogen	Fibrinogen-thrombin crosslinking	Low mechanical strength	Vascular tissue engineering, wound healing	Application in vascular grafts and heart tissue engineering
Bioinks with natural biomaterials	Hyaluronic Acid	Hyaluronic acid hydrogels	Crosslinking using UV or enzymatic methods	Fast degradation	Skin tissue engineering, cartilage repair	Injectable hydrogels for cartilage repair
Bioinks with natural biomaterials	Chitosan	Chitosan-alginate blends	Chemical crosslinking with glutaraldehyde	Limited solubility in physiological conditions	Wound healing, cartilage and bone tissue engineering	Chitosan blends for 3D printed scaffolds
Bioinks with natural biomaterials	Matrigel	Commercial Matrigel	Extrusion-based bioprinting, casting	High cost, batch-to-batch variability	Tumor modeling, organoid research, tissue regeneration	3D bioprinting for organoids and disease modeling
Bioinks with synthetic biomaterials	Poly(ethylene glycol) (PEG)	PEG-diacrylate (PEGDA)	UV or thermal crosslinking, photopolymerization	Limited biological functionality	Scaffold fabrication, soft tissue engineering	Functionalization of PEG for enhanced cell adhesion
Bioinks with synthetic biomaterials	Poly(lactic-co-glycolic acid) (PLGA)	PLGA nanoparticles, PLGA scaffolds	Electrospinning, 3D printing, solvent casting	Poor hydrophilicity	Drug delivery, bone tissue engineering	Development of hybrid PLGA bioinks with improved hydrophilicity
Bioinks with synthetic biomaterials	Polycaprolactone (PCL)	PCL scaffolds, PCL filaments	Fused deposition modeling (FDM), electrospinning	Low cell adhesion	Bone and cartilage tissue engineering, vascular grafts	Incorporation of bioactive molecules for enhanced cell proliferation
Bioinks with synthetic biomaterials	Polylactic Acid (PLA)	PLA scaffolds	Fused deposition modeling (FDM), extrusion-based printing	Brittleness, slow degradation	Bone regeneration, scaffold fabrication	Use of PLA composites for enhanced mechanical properties
Bioinks with synthetic biomaterials	Poly(vinyl alcohol) (PVA)	PVA hydrogels, PVA blends	Crosslinking using borate ions, freeze-thaw method	Limited biodegradability	Cartilage repair, tissue scaffolds	PVA-based hydrogels for cartilage tissue regeneration
Bioinks with natural and synthetic biomaterials	Alginate + GelMA	Alginate-GelMA composites	UV crosslinking, extrusion-based bioprinting	Limited mechanical strength	3D bioprinting for vascularized tissue engineering	Enhanced cell-laden bioinks for tissue vascularization
Bioinks with natural and synthetic biomaterials	Collagen + PEG	PEGylated collagen, Collagen-PEG hydrogels	UV crosslinking, enzymatic crosslinking	Costly, complex preparation	Skin tissue engineering, wound healing	PEGylation to improve stability and cell adhesion
Bioinks with natural and synthetic biomaterials	Chitosan + PCL	Chitosan-PCL scaffolds	Electrospinning, solvent casting	Poor solubility of chitosan, limited cell adhesion for PCL	Bone and cartilage tissue engineering	Functionalization of chitosan for enhanced bioactivity
Bioinks with natural and synthetic biomaterials	Hyaluronic Acid + PEG	PEGylated hyaluronic acid hydrogels	UV crosslinking, enzymatic crosslinking	Fast degradation of hyaluronic acid	Soft tissue engineering, cartilage repair	Injectable PEG-hyaluronic acid hydrogels for cartilage repair
Bioinks with natural and synthetic biomaterials	Alginate + PVA	Alginate-PVA hydrogels	Freeze-thaw method, extrusion-based bioprinting	Poor biodegradability of PVA	Tissue scaffolds, cartilage regeneration	Development of alginate-PVA composites for tissue engineering

#### Bioinks with natural materials

Hydrogels are generally too weak in mechanical strength to engineer tissues such as bone and cartilage. Due to this limited deficiency, biomaterials are very often incorporated with other agents in order to obtain functionality in hydrogels. In one case, alginate was combined with gelatin; hence, Alg-Gel was formed. It was observed that a pre-cross-linked alginate bioink was too fluid to print while temperature-controlled Alg-Gel has higher viscosity and consistency, hence improving the printability.^130^ High-pressure printing did not affect cell viability during in vitro tests with myoblasts. Another bioink was developed by Das et al. by mixing silk fibroin with gelatin and crosslinking with tyrosinase. The resulted bioink, which they called SF-G bioink, was tested using human nasal inferior turbinate tissue-derived mesenchymal stromal cells. Constructs revealed multilineage differentiation and tissue formation. Thermo-responsive agarose hydrogels were mechanically stiffened by incorporating type I collagen and were printed to investigate the resulting constructs’ osteogenic differentiation-supporting capabilities. Mesenchymal stem cell osteogenic differentiation was improved, and enhanced cell spreading with the maintenance of cell phenotype was demonstrated.^131^ Law et al. investigated the rheological properties, stability, structural behavior, and cell viability of hydrogels containing hyaluronic acid and methylcellulose. Their results showed that higher HAMC concentrations present the optimum for cell encapsulation.^132^

#### Bioinks with synthetic biomaterials

PEG has been one of the most commonly used synthetic biomaterials in 3D printing; however, the hydrophilic nature contributes to a loss of its structure-forming ability. Therefore, its use in mixtures with other synthetic materials is quite common, since that allows for an increase in mechanical properties. Joas et al. developed a hydrogel using PEG diacrylate with added anionic and cationic monomers such as 3-sulfopropyl acrylate and [2-(acryloyloxy)-ethyl]-trimethylammonium chloride, crosslinked visible light, adopting an extrusion-based printing process.^133^ This work was then extended by Xu et al., who synthesized a triblock copolymer composed of a diacrylated polycaprolactone and poly(ethylene glycol)-polycaprolactone (PCL-PEG-PCL) showing enhanced cellular support due to the increased survival rates.^134^ For cartilage constructs, a hydrogel of methacrylated pHPMA-lac combined with PEG was prepared, supplementing the previous formula with methacrylated hyaluronic acid in order to enhance its printability. A pH-sensitive, thermoresponsive hydrogel was prepared using methacrylated PEO-PPO-PEO showing reversible swelling-deswelling properties and fabricated by stereolithography.^135^ Larush et al. prepared a drug-loaded system, which consisted of acrylic acid monomer, polyethylene glycol diacrylate, and 2,4,6-trimethylbenzoyl-diphenylphosphine oxide (TPO) nanoparticles, displaying pH-responsive drug release properties.^136^

#### Bioinks with synthetic and natural biomaterials

Biocompatibility, cross-linking ability, mechanical strength, and thermal properties can be improved by combining natural and synthetic materials. GelMA is one of the most applied hybrid bioinks, which provides optimal cell proliferation, migration, and differentiation. GelMA is synthesized through a reaction involving the methacrylation of gelatin, where the former contributes biocompatibility and the latter contributes to greater mechanical strength. Duan et al. utilized gelatin methacrylate to fabricate trileaflet heart valves and better maintained human aortic valve interstitial cells, improving cell adhesion.^137^ Others blended chitosan with acrylamide to create a bioink suitable for digital light processing to fabricate 3D hydrogels whose biological and mechanical properties were enhanced for the construction of tissues and organs.^138^ Further, alginate and PLGA were used for the 3D printing drug delivery device.^139^

#### Bioinks with hydrogels and particles

Certain nanomaterials are known to improve the mechanical, chemical, and electrical properties of hydrogels. For instance, microparticles made from polyethylene glycol (PEG) were incorporated into cell-laden carboxymethyl cellulose (CMC) to enhance mechanical strength and increase viscosity. However, this led to a significant reduction in cell viability due to increased stress. To address challenges like shear thinning, self-healing, and adjusting mechanical properties, silicates are being incorporated into biomaterials.^140^ In one example, clay nanosheets were used to crosslink poly(N-isopropyl acrylamide), resulting in a hydrogel that stretched 1424% beyond its original length, while maintaining strength and durability.^141^ Hydroxyapatite, a naturally occurring component in bone tissue, is commonly applied in bone tissue engineering.^142^ The combination of alginate and hydroxyapatite (Hap) promoted chondrocyte secretion of calcified matrices through the porous structures formed by these materials.^143^ Tricalcium phosphate (TCP) is also used in the creation of bone tissue constructs and implants due to its ability to induce osteogenesis.^144^ In one study, a bioprinted shell of alginate hydrogel containing preosteoblast cells was used to extrude alpha-TCP paste, forming a bone tissue construct. This scaffold exhibited enhanced osteogenic differentiation as a result of the alpha-TCP content.^145^ Carbon-based nanomaterials, with their superior electrical and mechanical properties, are being utilized in neural and muscle tissue engineering. For example, GelMA and graphene bioinks were used to fabricate a precise structure, resulting in enhanced neural differentiation, suggesting potential applications for creating advanced nerve guidance systems.^146^

### Cell based bioinks

**Figure 6.**
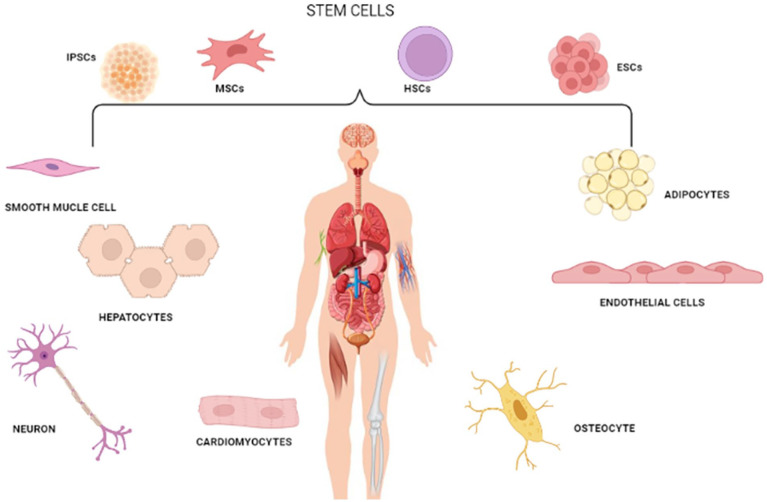
Cell-based bioinks.

Advanced technology has significantly improved the integration of a variety of cell types and biomaterials into a singular tissue construct through the use of tissue engineering and 3D printing techniques, as can be seen in Table 7 and Figure 6. To date, various bio-inks have been fabricated for different applications in regenerative medicine and tissue engineering by means of the layer-by-layer approach with cell-laden hydrogels.^147^ Some of the key considerations during bioprinting are the maintenance of high viscosity for a homogeneous cell suspension with structural integrity, high shear-thinning properties, and rapid gelation to support proper cell embedding. Various studies have been conducted with regard to skin bioprinting: for instance, the fabrication of skin tissue constructs comprising fibroblasts and keratinocytes to simulate the dermal and epidermal layers, respectively.^148^ The placing of 20 layers of fibroblasts and keratinocytes on a commercial acellular skin graft significantly enhanced proliferation and differentiation of cells. This study used a human-derived adipose tissue stem cell bioink for the fabrication of a dome-shaped construct with the aim of adipose tissue bioprinting. Implantation of the fabricated dome shape into mice has shown connective tissue remodeling, new formation of adipose tissue, and presence of standard markers related to adipogenic differentiation, hence indicating successful cell viabilities.

**Table 7.** Cell based bioinks.

Cell type	Examples	Technique/Process	Limitation	Application	Recent advances
Stem cell-based bioinks	Mesenchymal Stem Cells (MSCs), iPSCs, ESCs	Extrusion-based bioprinting, Inkjet bioprinting, Laser-assisted bioprinting	Cell viability and differentiation can be compromised during printing	Tissue regeneration, organ printing, cartilage repair	Development of encapsulation techniques for improving cell viability and differentiation
Immune cell-based bioinks	Macrophages, T cells, Dendritic Cells	Coaxial extrusion printing, Encapsulation techniques	Low cell survival rate in bioprinting processes	Cancer immunotherapy, wound healing	Enhanced immune cell bioprinting techniques for immunotherapy
Fibroblast-based bioinks	Dermal Fibroblasts, Gingival Fibroblasts	Extrusion-based printing, scaffold-based bioprinting	Limited cell proliferation in scaffolds	Skin regeneration, wound healing, dermal substitutes	Use of fibroblast-laden bioinks for developing complex skin tissue models
Endothelial cell-based bioinks	HUVECs, Blood Vessel Endothelial Cells	Extrusion-based printing, Coaxial extrusion for vessel creation	Difficulty in maintaining cell viability and forming functional blood vessels	Vascular tissue engineering, creation of microvascular networks	Advances in 3D bioprinting for the fabrication of functional microvascular networks
Muscle cell-based bioinks	Myoblasts, Skeletal Muscle Cells	Inkjet bioprinting, Laser-assisted bioprinting	Poor mechanical properties of muscle bioinks	Skeletal muscle regeneration, muscle tissue engineering	Development of hybrid muscle cell bioinks with improved mechanical properties
Neural cell-based bioinks	Neural Stem Cells (NSCs), Astrocytes, Schwann Cells	Extrusion bioprinting, Inkjet printing	Difficulty in maintaining functional neural networks	Neural tissue regeneration, spinal cord repair	Incorporation of bioactive molecules for enhancing neural cell proliferation and differentiation
Chondrocyte-based bioinks	Articular Chondrocytes, Nasal Chondrocytes	Coaxial extrusion printing, scaffold-based bioprinting	Limited proliferation and viability	Cartilage repair, osteoarthritis treatment	Advances in chondrocyte-laden bioinks for cartilage regeneration and repair
Hepatocyte-based bioinks	Primary Hepatocytes, Hepatoma Cells	Extrusion-based printing, scaffold-free bioprinting	Hepatocytes lose their functions rapidly after printing	Liver tissue engineering, drug screening, disease modeling	Development of functional liver tissues using hepatocyte-laden bioinks
Cardiomyocyte-based bioinks	iPSC-derived Cardiomyocytes	Extrusion-based printing, scaffold-based bioprinting	Limited long-term viability and contractile functionality	Cardiac tissue engineering, heart patches for myocardial infarction treatment	Recent advances in creating functional heart tissues with cardiomyocyte-laden bioinks
Adipose cell-based bioinks	Adipose-derived Stem Cells (ASCs), Mature Adipocytes	Inkjet printing, Extrusion-based printing	Difficulty in maintaining adipose tissue structure and function	Soft tissue regeneration, breast tissue reconstruction	Development of vascularized adipose tissues for soft tissue engineering

In addition, a number of engineered skeletal muscle constructs have been fabricated. These were designed to model native tissue organization. Bioprinting, using the ITOP system, was employed to align and stretch muscle fibers as one approach to model native tissue architecture.^149^ For vascular systems, techniques using bioprinting have generated functional blood vessels and vascular networks. A model was fabricated with human aortic SMCs, human aortic ECs, and human dermal fibroblasts. Cells were bioprinted on agarose templates as a base, assembled into cell spheroids within cylindrical structures mimicking blood vessels. It also covers some advances toward the fabrication of 3D liver models with the use of cell-encapsulated constructs. One of the studies printed hepatocyte-laden alginate hydrogel bioink within a microfluidic chamber and thereafter expressed continuous cell growth and proliferation as well as liver-specific functionality. Even though research is still in its beginning, stem cells are being used as bioprinting bioinks. Mesenchymal stromal cells are one of the most common used because they are easy to culture and expand.^150^ MSCs could be taken from a number of tissues such as bone marrow and adipose tissue. Bioprinted MSCs put together into a template that eventually supported vascularized bone ingrowth. The constructs also contained alginate, RGD motifs for cell adhesion, and a PCL supporting network.^151^ Another group produced a bone niche via inkjet printing with ceramic nanoparticles embedded within PEG in addition to printing MSCs with hydroxyapatite for bone development.^152^ In the case of neurodegenerative diseases, the application of bioprinting of neural tissues using NSCs is under consideration. Many studies have revealed that NSCs have huge potential as one of the candidates in bioprinting. In this regard, the printing of murine NSCs with polyurethane gel showed higher neurotrophic genes than TCPs.^153^ Human NSCs are also printed with agarose-alginate and carboxymethylcellulose showing good cellular viability and the ability to follow differentiation protocols even after printing. Due to their competency of unlimited self-renewal, PSCs represent a perfect candidate for the fabrication of renewable human tissues. The major sources of PSCs are hiPSCs and human ESCs.^154^ In order to overcome the fragility of hESCs during bioprinting, a valve-based printing technique was developed. That technique was further utilized in order to fabricate and differentiate hiPSCs into functional hepatocytes.^155^

## Regulatory affairs in bioink

The regulatory mechanisms of bioink in 3D bioprinting are very complex and constantly evolving. Safety, efficacy, and ethical considerations require full vigilance. Regulators do their best to try to modify frameworks that can keep pace with innovation as technology advances (Tables 8 and 9). In the United States, regulation of bioinks and bioprinted products is mainly governed by the FDA. Depending on their composition and intended use, such products could be considered a medical device, a biologic, or a combination product. Medical devices such as acellular scaffolds would fall under the category overseen by the CDRH, while bioinks that contain live cells would be considered biologics and fall under the auspices of the CBER. In the same line, the EMA in the European Union does something similar to bioinks under its advanced therapy medicinal products for cell and gene therapies. Japan’s PMDA developed progressive frameworks allowing conditional early approval under certain conditions for certain regenerative medicinal products.

**Table 8.** Regulatory bodies of different countries responsible for medical products regulation.

Country	Regulatory body	Key feature
United States	Food and Drug Administration (FDA)	Stringent pre-market approval process ensures safety and efficacy of medical products.
European Union	European Medicines Agency (EMA)	Centralized authorization process for medicines facilitates access across EU member states.
United Kingdom	Medicines and Healthcare products Regulatory Agency	Rapid approval pathways for innovative medical products support timely access for patients.
Canada	Health Canada	Comprehensive post-market surveillance system for monitoring and addressing safety concerns.
Japan	Pharmaceuticals and Medical Devices Agency (PMDA)	Priority review system expedites approval for innovative drugs, addressing unmet medical needs.
Australia	Therapeutic Goods Administration (TGA)	Robust evaluation of medical devices conformity to ensure safety and performance standards.
Brazil	Agência Nacional de Vigilância Sanitária (ANVISA)	Risk-based regulatory assessment streamlines approval processes for low-risk medical products.
India	Central Drugs Standard Control Organization (CDSCO)	Accelerated approval pathways for essential medicines addressing public health needs efficiently.
China	National Medical Products Administration (NMPA)	Emphasis on international collaboration and alignment with global standards to enhance product quality and safety.
South Africa	South African Health Products Regulatory Authority	Transparent and collaborative regulatory processes fostering innovation while ensuring public health protection.
Russia	Federal Service for Surveillance in Healthcare	Implementation of strict requirements for clinical trials and thorough evaluation of medical products before approval.
Singapore	Health Sciences Authority (HSA)	Robust pharmacovigilance system for monitoring adverse drug reactions and ensuring continued safety post-market.
Mexico	Comisión Federal para la Protección contra Riesgos Sanitarios	Expedited approval process for medical devices with international certifications, facilitating market access.
Switzerland	Swissmedic	Collaboration with international regulatory agencies for mutual recognition of approvals, streamlining market access.
Argentina	Administración Nacional de Medicamentos, Alimentos y Tecnología Médica	Periodic revaluation of registered medicines ensures continued safety and efficacy.
Saudi Arabia	Saudi Food and Drug Authority (SFDA)	Adoption of electronic submission systems and digital platforms for regulatory processes, enhancing efficiency and transparency.
Israel	Ministry of Health, Pharmaceutical Division	Regulatory support and incentives for research and development of orphan drugs and rare diseases treatments.
Nigeria	National Agency for Food and Drug Administration and Control	Stringent measures to combat counterfeit medicines and substandard medical products.
Thailand	Food and Drug Administration Thailand (FDA Thailand)	Integration of traditional medicine and herbal products into regulatory frameworks to ensure safety and efficacy.
Indonesia	National Agency of Drug and Food Control (BPOM)	Collaboration with international regulatory agencies to harmonize standards and facilitate trade of medical products.
Vietnam	Ministry of Health, Drug Administration of Vietnam	Development of risk-based regulatory approaches to expedite approval processes for urgently needed medical products.
Malaysia	National Pharmaceutical Regulatory Agency (NPRA)	Implementation of Good Manufacturing Practice (GMP) guidelines to ensure quality standards for pharmaceutical manufacturing.
South Korea	Ministry of Food and Drug Safety (MFDS)	Utilization of advanced technologies such as artificial intelligence and big data analytics for drug safety monitoring.
Philippines	Food and Drug Administration Philippines (FDA Philippines)	Establishment of a Unified Licensing System for efficient processing of licenses and permits for pharmaceuticals and medical devices.
Turkey	Turkish Medicines and Medical Devices Agency (TITCK)	Implementation of a risk-based inspection system to prioritize inspections of pharmaceutical manufacturing facilities.
Pakistan	Drug Regulatory Authority of Pakistan (DRAP)	Strengthening of pharmacovigilance systems for improved monitoring and reporting of adverse drug reactions.
Ukraine	State Expert Center of the Ministry of Health of Ukraine (SEC)	Implementation of reforms to harmonize regulatory standards with international practices, facilitating market access.
Chile	Instituto de Salud Pública de Chile (ISP)	Provision of technical assistance and capacity-building programs to support compliance with regulatory requirements.
Peru	General Directorate of Medicines, Supplies, and Drugs (DIGEMID)	Collaboration with international regulatory agencies to strengthen regulatory capacity and promote access to safe and effective medical products.
Colombia	Instituto Nacional de Vigilancia de Medicamentos y Alimentos (INVIMA)	Implementation of electronic submission systems and digital platforms for regulatory processes to enhance efficiency and transparency.

**Table 9.** Regulatory aspects in bioinks for 3D bioprinting.

Regulatory aspect	Details
Regulatory Bodies	FDA (USA), EMA (EU), PMDA (Japan), ISO, ASTM
Classification Systems	Medical Devices, Biologics, Combination Products
Key Standards & Guidelines	ISO 10993 (Biocompatibility), FDA’s Premarket Approval (PMA) and 510(k), EMA’s ATMPs framework
Biocompatibility Testing	In vitro cytotoxicity tests, In vivo animal studies to test immune response, genotoxicity, and tissue integration
Sterility Requirements	Strict GMP compliance: Use of cleanrooms, Endotoxin testing, and validated sterilization methods (filtration, irradiation)
Mechanical Integrity Testing	Mechanical testing to assess tensile, compressive strength, and degradation profiles to ensure safety and efficacy
Preclinical Studies	Preclinical testing in small and large animal models to assess biocompatibility and functionality in biological systems
Clinical Trials Phases	Phase I (safety), Phase II (efficacy and dosing), Phase III (confirmation and large-scale testing)
GMP (Good Manufacturing Practices)	ISO 13485 certified quality management systems, GMP requirements for sterility, consistency, and documentation
Ethical Considerations	Use of stem cells (ESCs, iPSCs) involves ethical issues; informed consent for cell donors and 3Rs principles in animal testing
Challenges in Approval	Challenges include lack of established guidelines for customized bioprinted products, complex bioink compositions, and regulatory delays
Recent Regulatory Advances	FDA’s Emerging Technologies Program, RMAT designation for expedited approval of life-saving therapies
Global Harmonization	ISO and ASTM work toward global standards for bioink testing and production; regulatory collaboration across borders
Case Study 1: FDA Approval of OsteoFab	FDA cleared 510(k) approval for a 3D-printed PEKK cranial device, demonstrating reliability of customized implants
Case Study 2: Bioprinted Skin by CSIC & BioDan	Clinical trials on burn patients; uses bioinks made of plasma, fibroblasts, and keratinocytes; success in wound healing applications
Case Study 3: CELLINK Bioinks	CELLINK’s bioinks for research and clinical purposes follow ISO 13485 and GMP standards; overcoming challenges in sterility and scaling production

One of the major challenges in regulating bioinks involves classification. Accordingly, bioinks range from simple hydrogel materials, which could be classified as medical devices, to complex biologics that incorporate live cells, proteins, or growth factors. Medical devices, therefore, will have less stringent approval processes, usually through Premarket Approval and 510(k) clearance, depending on the level of risk associated with them. Where biologics, on the other hand, have to go through more stringent testing in regard to safety, purity, and potency under Biologics License Applications. For combination products-combination of both biological parts and devices-the regulatory agencies like FDA designate a lead center according to a product’s primary mode of action, hence making the regulatory process complicated. Safety, biocompatibility, sterility, mechanical integrity-the list goes on concerning the regulatory pathways which bioinks must follow.

Testing the biocompatibility of the bioinks would be performed to check whether the material, in vivo, would create adverse immune responses or toxicity. This would mean a combination of in vitro cytotoxicity screening tests and in vivo studies investigating systemic toxicity and immune reactions. The other major concern is sterility. Bioinks, in particular those with living cells, have to be fabricated and processed under aseptic conditions to avoid contamination. This presupposes strict GMP, including cleanrooms, sterilization methods, and high-stringency endotoxin testing. The regulatory authorities also expect that the bioinks have adequate mechanical properties, especially for applications at load-bearing tissues such as bone or cartilage. Mechanical testing helps confirm that printed structures bear physiological stresses with minimal degradation of functionality. Another huge regulatory challenge is ensuring the safety and efficacy of bioinks; toxicity and functional performance must be tested in preclinical animal models of bioinks in biological systems before clinical use.

These large animal systems are selected due to their close anatomical and physiological relationship to humans. After bioinks have completed preclinical trials, they progress to clinical trials in Phase I, where the bioinks are utilized on a small group of humans in order to test for safety, followed by Phase II and III trials, which test the efficacy of the treatment, with further monitoring for side effects. The regulatory bodies also demand that the manufacturers ensure a very high level of quality control, ensuring standards such as GMPs that may be inclusive of strong quality management systems, equipment calibration, and personnel training. There are a couple of original challenges in the regulatory landscape for 3D bioprinting and bioinks: indeed, one of the greatest appears to be the customization of bioprinted constructs. Due to the fact that most 3D bioprinting makes patient-specific implants or tissues, it is difficult to achieve standardization.

The control bodies may permit such personalized treatments under exemptions or compassionate use pathways, but the establishment of consistent guidelines with respect to the regulation of such custom products has yet to be resolved. Besides, the rapid pace with which the technology is evolving in bioprinting is abreast with that of regulating frameworks, delaying product approvals. If this were not enough, there is also the material complexity of the bioinks-most often a combination of natural, synthetic, and biological components put together. But complications add another layer of complexity to the approval process, such as how these various components need to be stringently examined by regulatory agencies for their interactions. As a solution to these problems, regulatory agencies begin to adapt more flexible criteria. To help companies work through regulatory requirements for their innovative bioprinting technology, the Food and Drug Administration in the United States has one program: the Emerging Technologies Program. Likewise, the Regenerative Medicine Advanced Therapy designation provides expedited pathways for approval to those products of promising therapy for serious or life-threatening conditions. The programs balance promotion of innovation with safeguarding safety.

Another direction is the standardization of testing methodologies of bioinks and bioprinted constructs. Now, international organizations such as ISO and ASTM International are developing global standards regarding manufacturing and testing of bioinks. Elaboration of such unified standards will promote harmonization of regulatory pathways across different regions and allow international collaboration and market access. Interlaboratory studies are also being conducted to validate these testing methods and ensure that they are reliable, reproducible across different settings. Bioink development, in particular, will have to consider a number of important ethical considerations when fabricating with live cells or even stem cells.

ESCs raise ethical concerns, while iPSCs reprogram adult cells into serving as a less-controversyael substitute. In general, regulatory agencies require that cell-based products conform to strict ethical guidelines; these include taking proper informed consent from the donors. Another ethical concern is animal testing, where many researchers work in order to follow the 3Rs principle: Replacement, Reduction, Refinement. This involves minimizing the usage of animals in preclinical tests.^156–162^

### Case studies pertaining to regulatory affairs in bioinks for 3D printing

**a)** **Case Study 1:** FDA Approval of OsteoFab Patient-Specific Cranial Device by Oxford Performance Materials The OsteoFab Patient-Specific Cranial Device (OPSCD) is one of the pioneering 3D printed implants to gain FDA 510(k) clearance as a Class II medical device. Developed by Oxford Performance Materials (OPM), the device is a patient-specific cranial implant made from polyetherketoneketone (PEKK), a high-performance thermoplastic polymer. This case demonstrates how regulatory frameworks for 3D printed medical devices have evolved and illustrates the challenges and solutions encountered during the approval process.

*Development and Technology*: The OPSCD was created using advanced 3D printing technology that allowed for the customization of cranial implants based on patient-specific anatomical data, such as CT scans. The material used, PEKK, is biocompatible, highly resistant to chemicals, and possesses mechanical properties similar to human bone, making it an ideal candidate for cranial implants. The process of 3D printing allows for precise customization, ensuring that the implant fits the unique contours of a patient’s skull.

*Regulatory Pathway*: To achieve FDA approval, OPM followed the 510(k) clearance process, which requires demonstrating that the device is substantially equivalent to a legally marketed predicate device. This pathway is typically less stringent than a full Premarket Approval (PMA), but still requires extensive testing for biocompatibility, mechanical integrity, and safety. OPM conducted a battery of preclinical tests, including mechanical testing to ensure that the PEKK material could withstand physiological forces and biocompatibility studies to assess any adverse tissue responses. The FDA also required that OPM demonstrate the reproducibility and consistency of their manufacturing process, as patient-specific devices must meet high precision standards.

*Challenges and Solutions*: One of the main challenges faced during the approval process was the high level of customization inherent in 3D printed implants. Because each implant is unique to the patient, it was difficult to apply standardized regulatory requirements. However, OPM worked closely with the FDA to demonstrate that despite the customization, their 3D printing process could reliably produce high-quality implants with consistent mechanical and biological properties. The company also developed rigorous quality control measures, including detailed documentation of the entire manufacturing process for each individual implant.

*Outcome*: The FDA’s 510(k) clearance of the OPSCD marked a significant milestone in the medical device field, showing that 3D printed patient-specific implants could meet stringent regulatory standards. Since its approval, the OPSCD has been used in numerous patients, demonstrating the efficacy and safety of 3D printed cranial implants for reconstructive surgery. The success of this case has paved the way for further innovations in 3D printed medical devices and has helped establish regulatory frameworks for future bioprinted products.

**b)** **Case study 2:** Bioprinted Skin by Spanish National Research Council (CSIC) and BioDan Group. The development of bioprinted human skin by the Spanish National Research Council (CSIC), in collaboration with BioDan Group, represents a breakthrough in the use of 3D bioprinting for regenerative medicine. This project focuses on creating functional human skin using bioinks composed of plasma, fibroblasts, and keratinocytes, with applications ranging from burn treatment to pharmaceutical testing. The bioprinted skin is the first of its kind to undergo clinical trials, demonstrating the potential of bioinks in real-world medical applications.

*Development and Technology*: The bioprinted skin project utilized bioinks made from a combination of plasma and human fibroblasts (cells that generate connective tissue) and keratinocytes (the predominant cells in the outer layer of the skin). The technology allowed for the precise deposition of these cells layer by layer to recreate the structure of human skin, including both the dermis and epidermis. The key innovation in this project was the ability to produce skin with characteristics very similar to natural human skin in terms of texture, elasticity, and function.

*Preclinical Testing and Challenges*: Before moving to clinical trials, the bioprinted skin underwent extensive preclinical testing to assess its safety and functionality. Animal models were used to demonstrate that the bioprinted skin could integrate with host tissue, promote wound healing, and maintain functionality over time. One of the major challenges encountered was ensuring the sterility of the bioink and bioprinting process, as contamination could compromise the viability of the cells and lead to infections. Additionally, maintaining the viability and functionality of fibroblasts and keratinocytes during and after the bioprinting process was a significant hurdle, as cells can experience stress during extrusion and post-printing processes. Researchers developed special protocols to ensure that the cells maintained their viability and proliferative capabilities after printing.

*Regulatory Pathway*: Once the preclinical tests confirmed the biocompatibility and efficacy of the bioprinted skin, the project moved to clinical trials. The Spanish Agency of Medicines and Medical Devices (AEMPS), which regulates pharmaceuticals and medical devices in Spain, approved the trials after reviewing the extensive preclinical data. The clinical trials involved burn patients who received bioprinted skin grafts to evaluate how well the bioprinted constructs integrated into their natural tissue. Key endpoints in these trials included healing time, reduction in scarring, and overall skin function.

*Outcome*: The trials are ongoing, but early results have been promising, showing that bioprinted skin is a viable treatment for burn wounds and other skin injuries. The ability to create large quantities of functional human skin in the lab could revolutionize treatments for severe burns and open the door for applications in cosmetic surgery, pharmaceutical testing, and disease modeling. This project serves as a critical case study for how bioinks can be successfully developed and regulated for clinical use, overcoming challenges related to cell viability, biocompatibility, and manufacturing scale.

## Regulatory aspect pertaining to use of bioinks in medical devices

### Regulatory landscape and classification challenges

Bioinks, designed for use in medical devices such as scaffolds for tissue regeneration or organ printing, face a complex, and evolving regulatory landscape. A primary challenge lies in their classification, as bioinks straddle the boundary between medical devices, biologics, and combination products.^163^ Regulatory agencies such as the FDA (Food and Drug Administration) in the United States and the EMA (European Medicines Agency) in Europe often have distinct pathways and requirements for these categories. The challenge amplifies when bioinks are modified with living cells or therapeutic agents, as this blurs the distinction between a scaffold and a tissue-engineered product.^164,165^ For instance, acellular bioinks may be regulated as Class II devices under the FDA’s 510(k) pathway, whereas cell-laden bioinks could be classified as combination products requiring extensive clinical trials. This disparity in regulatory pathways creates hurdles for manufacturers aiming to align product development with market requirements, potentially delaying commercialization.^166^

### Quality control and standardization of bioinks

The inherent variability in bioink composition poses significant regulatory challenges, particularly in ensuring reproducibility and safety. Bioinks often incorporate natural polymers, synthetic materials, and biologically active components, each with unique degradation profiles and biocompatibility characteristics.^167^ For instance, bioinks derived from decellularized extracellular matrices may exhibit batch-to-batch variability due to differences in source tissues or processing methods. Regulatory bodies require stringent quality control protocols to ensure consistency, but the lack of standardized methods for characterizing bioinks, such as rheological properties, cell viability support, and degradation kinetics, complicates compliance.^56^ Furthermore, the interaction of bioinks with printing technologies introduces additional variables, such as thermal or shear stress during extrusion, which could impact the final product’s performance. Establishing universal standards for bioink testing and certification remains a critical need to bridge these gaps.^168^

### Long-term safety and clinical translation concerns

The regulatory approval of bioinks used in medical devices hinges on comprehensive preclinical and clinical testing to evaluate their biocompatibility, biofunctionality, and long-term safety. However, the long-term effects of bioinks in vivo remain underexplored, particularly concerning their degradation products and immunogenic response.^169^ For example, while synthetic bioinks may offer controlled degradation rates, their by-products could provoke localized toxicity or inflammatory responses over extended periods. Conversely, natural bioinks may elicit immune reactions depending on their source and purification level. The challenges extend to the clinical translation of bioink-enabled devices, where the complexity of manufacturing processes under Good Manufacturing Practice (GMP) conditions adds an additional layer of difficulty.^170^ Regulatory agencies require robust data demonstrating the bioink’s stability, sterility, and performance under physiological conditions, which can be resource-intensive for developers. Addressing these concerns requires interdisciplinary collaboration and the development of advanced testing frameworks to facilitate the safe integration of bioinks into clinical applications.^171^

## Conclusion

Three-dimensional bioprinting represents a promising state-of-the-art technology with extensive capabilities of fabricating tissues and organs using cell-laden materials. The present review covers major variants of biomaterials, which are typically used to fabricate bioinks: natural, synthetic, and hybrid polymers. Each class exhibits advantages either in their pure form or in combination with other biomaterials to enhance the efficiency of a particular bioprinting methodology. Natural bioinks, based on biomaterials such as collagen, gelatin, and silk, are capable of accurately reproducing the biocompatible ECM of native tissues and cell attachment and proliferation within it. Protein-based bioinks may naturally imitate the ECM for tissue engineering through the enhancement of cell growth and the eventual formation of the ECM. The use of dECM combined with 3D bioprinting methods develops a promising methodology in the repair of tissues and organs, the creation of organoids, construction of disease models, and the study of disease mechanisms. This may alter the natural configuration of proteins, which influences cell-matrix interaction and can elicit an immune response capable of causing problems in regeneration after implantation.

Future studies need to focus on the development of native-state protein bioinks that closely mimic natural environments and enhance mechanical strength in order to have precise scaffold fabrication. Immune response-ensuring biocompatibility-overcoming will become the main critical issues for successful TE applications of these bioinks. Moreover, biomaterials have several vulnerabilities, such as poor mechanical strength and possible immune responses. While these bioinks face challenges, they offer a promising platform for bioprinting by creating conducive environments for cells while necessitating strategies to overcome their limitations and enhance effectiveness. Synthetic bioinks, made via chemical synthesis without natural biomaterials, are usually based on synthetic polymers such as polyethylene glycol (PEG) or polycaprolactone (PCL). These are designed to possess specific properties desirable in bioprinting. Hybrid bioinks are prepared by the combination of natural and artificial materials to derive a benefit from both. For instance, alginate is usually mixed with synthetic polymers like PEG, which enhances its printability and mechanical properties. Such hybrid bioinks can be post-formulated with other additives like nanoparticles or growth factors for beyond cell support in promoting additional functional capabilities in tissues. They offer advantages with regard to tunable mechanical properties, increased printability, and even the possibility of adding bioactive agents for controlling cellular behavior and tissue engineering. However, while these inks have their advantages, perfecting formulation and cross-linking is an upcoming challenge in striving for properties and biocompatibility. Further research is Further research is needed to realize the full potential of synthetic bioinks and hybrid ones in various bioprinting applications related to tissue engineering, drug delivery, and regenerative medicine. Besides the traditional and widely applied cell-laden bioinks, there are exciting options with the use of bioinks that come from basic units of life, the extracellular matrix, decellularized tissues, and even cell aggregates. Cell-derived bioinks are in a leading position and can print complex forms of these materials, including those with viscosities that drop under stress, in a layer-by-layer fashion that allows progressive solidification of the printed structures. On the other hand, though direct cell printing boasts a number of advantages, only relatively small-sized constructs have so far been printed due to the large volume of cells required. Besides being printable into defined shapes, such bioinks need sufficient biocompatibility, adequate mechanical strength, and controllable degradation over a specific period for implantation. The optimization of the properties of bioinks for printability is important at all different stages of gelation for structural precision, shape fidelity, and cell viability. In fact, successful bioprinted tissues, as effective biological substitutes, can closely physically resemble natural tissues. Incorporating machine learning (ML) and artificial intelligence (AI) into the process of developing novel bioinks is a step in the right direction. Optimization of viscosity, elasticity, yield stress, and biological functioning are some of how these technologies improve the qualities of bioinks for use in 3D bioprinting. It is machine learning that improves bioink formulations by studying mechanical qualities, while artificial intelligence speeds up the development of new materials by determining the best compositions.

Furthermore, machine learning and artificial intelligence make it possible to create multifunctional bioinks that take the form of genuine tissues by combining bioactive compounds and materials that are sensitive. The use of artificial intelligence allows for the customization of bioinks for certain tissues, such as cartilage or vascular structures, and it also permits patient-specific applications, which improves therapeutic results in regenerative medicine and bioprinting.

By making novel bioinks, biomedical engineers are getting closer to making such structures combining a number of ideal features that would closely emulate native tissues for the purposes of regeneration, which in turn will also improve their therapeutic outcome. With the fast-growing research field in 3D bioprinting, its potential will further increase, leading to significant gains in science, and better healthcare for the patients.
